# Evolutionary algorithms converge towards evolved biological photonic structures

**DOI:** 10.1038/s41598-020-68719-3

**Published:** 2020-07-21

**Authors:** Mamadou Aliou Barry, Vincent Berthier, Bodo D. Wilts, Marie-Claire Cambourieux, Pauline Bennet, Rémi Pollès, Olivier Teytaud, Emmanuel Centeno, Nicolas Biais, Antoine Moreau

**Affiliations:** 1 Université Clermont Auvergne, CNRS, Sigma Clermont, Institut Pascal, 63000 Clermont-Ferrand, France; 2 TAO, Inria, LRI, Université Paris Sud CNRS UMR 6823, Orsay Cedex, France; 3 Adolphe Merkle Institute, University of Fribourg, Chemin des Verdiers 4, 1700 Fribourg, Switzerland; 4 Facebook AI Research, 6 rue Menars, 75000 Paris, France; 5 Graduate Center of CUNY and Department of Biology, CUNY Brooklyn College, New York, NY 11210 USA

**Keywords:** Biophotonics, Computer science, Computational science, Photonic crystals

## Abstract

Nature features a plethora of extraordinary photonic architectures that have been optimized through natural evolution in order to more efficiently reflect, absorb or scatter light. While numerical optimization is increasingly and successfully used in photonics, it has yet to replicate any of these complex naturally occurring structures. Using evolutionary algorithms inspired by natural evolution and performing particular optimizations (maximize reflection for a given wavelength, for a broad range of wavelength or maximize the scattering of light), we have retrieved the most stereotypical natural photonic structures. Whether those structures are Bragg mirrors, chirped dielectric mirrors or the gratings on top of Morpho butterfly wings, our results indicate how such regular structures might have spontaneously emerged in nature and to which precise optical or fabrication constraints they respond. Comparing algorithms show that recombination between individuals, inspired by sexual reproduction, confers a clear advantage that can be linked to the fact that photonic structures are fundamentally modular: each part of the structure has a role which can be understood almost independently from the rest. Such an *in silico* evolution also suggests original and elegant solutions to practical problems, as illustrated by the design of counter-intuitive anti-reflective coatings for solar cells.

## Introduction

Nature features a diversity of photonic architectures producing the most vivid optical effects^1–3^. Regularly alternating chitin and melanin layers constitutes, for instance, a very efficient way to reflect light, giving the structure a colored metallic appearance. Such structures can be found on the cuticle of many insects, including common flies (e.g. *Lucilia sericata*) or beetles (e.g. *Chrysolina americana* or *Asphidomorpha tecta*). These ubiquitous structures have been optimized through natural evolution during millions of years, which suggests that they provide an evolutionary advantage to all the animals. While such dielectric, multilayered mirrors^4^ are relatively simple, more complicated architectures can be found in many animals. Most famous are probably the nano-sized christmas-tree-like ridge structure of *Morpho* butterfly wing scales^5,6^. These nano-sized christmas-trees, assembled of transparent chitin, are responsible for the bright iridescent blue color of the *Morpho rethenor* wings which has made the butterfly so famous.

Numerical optimization has been extensively used in photonics for the last decades, especially in the context of optical filters^7,8^ for their technological importance—the multiplexers used to increase the bandwidth of optical fibers rely on such multilayered structures. By ‘simply’ choosing the thicknesses of transparent layers with alternating refractive index (RI), it is possible to design and manufacture virtually any kind of optical filter^9^. Efficient algorithms have been developed to solve precisely this kind of inverse problem: finding the right geometrical (thicknesses) and optical (RI) parameters of the structure to obtain the desired optical response. With the development of more versatile simulations tools in optics and the increase in the available computational power^10^, numerical optimization has been applied to more complicated problems, producing complex designs with interesting performances^11–15^. However, we underline that the most successful approaches to date^9,15^ do not rely on standard global optimization techniques. Attempts using early genetic or specifically designed algorithms^16–18^ did not provide sufficiently efficient designs to warrant their further use. Finally, except for one remarkable case where some regularity seemed to spontaneously emerge^19^, none of the structures obtained by numerical optimization seem to possess the regularity of natural structures^15^. Given the fundamental differences between naturally occurring structures and artificially optimized designs, there is still a knowledge gap as to why natural structures are regular or periodical in a seemingly different way than the fabricated ‘ideal’ structures and whether such a regularity is imposed for optical reasons.

Suspecting these structures occur mainly for optical reasons^19^, we here apply state-of-the-art evolutionary algorithms to problems of increasing complexity. We show that solutions that resemble natural photonic architectures can be retrieved with this class of algorithms. Each problem we study is defined by a quantity to minimize or maximize, like the reflectance or the scattering, and constraints, for instance on the values of the refractive index of the materials. Changing constraints make specific features emerge which are present in natural architectures, thus allowing to understand their role precisely. Furthermore, we show then how this method can be applied to key problems in the optical sciences, e.g. enhancing the absorption of light in solar cells, to generate regular and understandable devices.

To perform the optimizations, we have employed five different, state-of-the-art algorithms^20–24^ that seem to be best suited for our setting, i.e. complex real-world problems for which little is known a priori from a mathematical point of view. Evolutionary algorithms^20–24^ are computational trial-and-error algorithms that aim at finding optimal solutions to well posed mathematical problems while being inspired by evolutionary processes. In general, evolutionary algorithms consider a population of individuals, where each individual corresponds to a potential solution with well defined geometrical and optical parameters. An objective function allows to rate the fitness of a solution/individual: the lower the objective function, the better the solution and thus the “fitter” the corresponding individual. The population then evolves: while individuals that are not “fit” enough and present too high an objective function value are eliminated, new individuals are subsequently created, for example by combining the geometrical and optical characteristics of better individuals. Through this *in silico* evolution, the average fitness of the individuals increases and hopefully leads to the best possible solution to the problem posed, the so-called *optimal* solution that minimizes the objective function.

The algorithms differ in their original inspiration (see Supplementary Information for more details). The first ($1+1-{\text {ES}}$) is inspired by the evolution of bacteria, with local mutations taking a central place^24^; the second (Differential Evolution^21^) is (remotely) inspired by the evolution process of sexual selection and includes recombination between successful individuals; the last ones are less related to evolution. Particle Swarm Optimization^22^ is inspired by the behavior of swarms, while Covariance Matrix Adaptation and Nelder–Mead^20^ are more artificial algorithms based on profound mathematical considerations. All of them can be considered as global optimization methods, aiming at finding the true optimal solution in the whole landscape. This is not the case for gradient-based methods, which have spread recently in the photonics community^15^ and which are considered local—they start with an arbitrary solution and improve it as far as possible, and are thus not meant to find the global optimum.

## Retrieving naturally occurring photonic structures

We first begin by considering multilayers of transparent materials, the simplest case of a one-dimensional photonic structure^2^, to investigate whether the algorithms are able to reproduce the regular structures of nature.

### Emergence of Bragg mirrors

The objective function is first defined so that the algorithms simply maximize the reflection coefficient of the structure for a given wavelength of the incident light—and computed using a freely available simulation tool for electromagnetic optics^25^. The algorithms are free to modify the thicknesses and the RI of the individual layers. For several numbers of layers ranging from 4 to 40, we run up to 100 optimizations for each algorithm with a budget of $10^4$ evaluations of the objective function (and thus the same number of new individuals). Each optimization takes only a few seconds when computed on a common desktop PC, using a single core. Dielectric mirrors in nature consist of high index layers (circa 1.7), predominantly made of melanin, comprised in a matrix of cuticular chitin and these usually begin with chitin, presenting the lowest RI (circa 1.55)^26^. The only constraint we impose is thus that the RI has to be in a given range of 1.4 to 1.7, typical for organic materials. The lower bound we impose here is actually slightly lower than in nature. Importantly, we have checked that our results do not depend on the refractive index range which is chosen by obtaining similar results for the naturally occurring index contrast (1.55–1.7), as well as for a higher one, more typical of technological situations (1.45–2.35^7^, see Supplementary).

**Figure 1:**
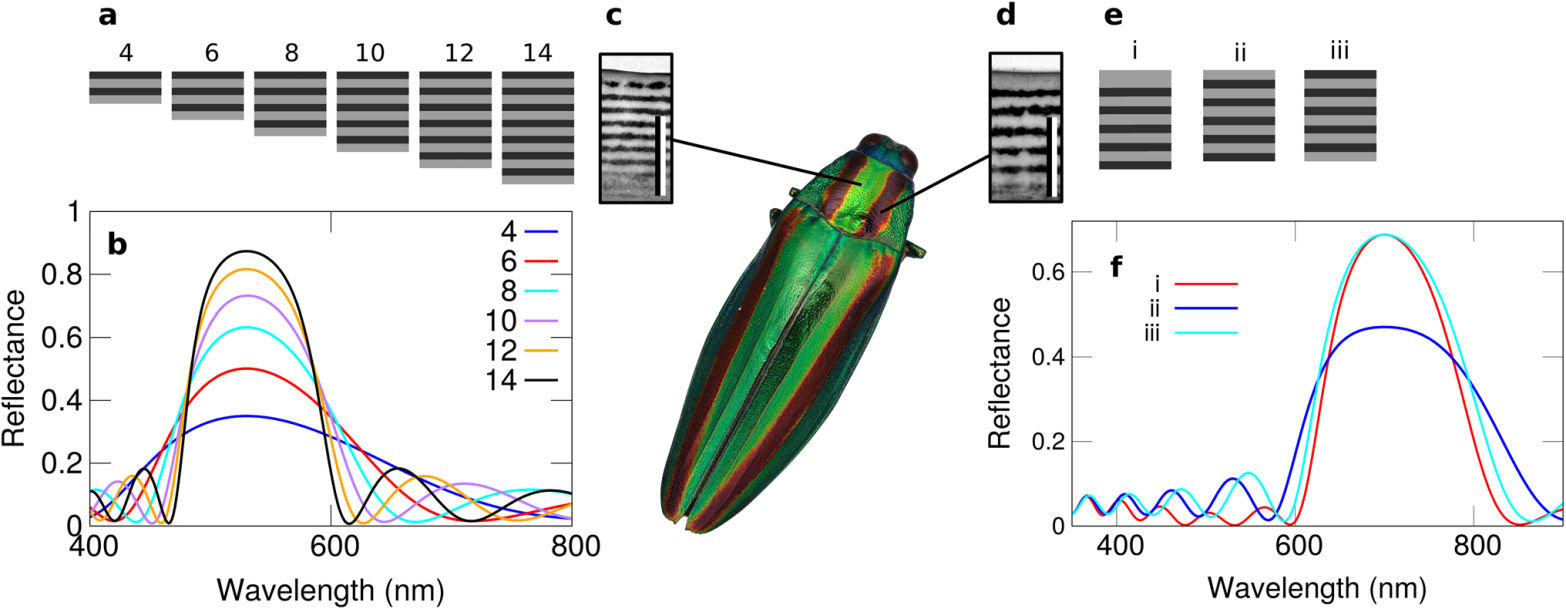
Retrieving dielectric mirrors through optimization. (**a**) Solutions obtained through optimization for different numbers of layers, ranging from 4 to 14 and drawn using Octave^28^. (**b**) Associated reflectance spectrum showing a higher reflection in the green part of the spectrum, around 530 nm. (**c,d**) TEM images of the cuticular surface structure of the Japanese Jewel beetle, *Chrysochroa fulgidissima*^29^ (green and purple part of the elytron, respectively), bar: 1 μm. (**e**) Dielectric mirrors beginning with the lower index with a $\lambda /2$ (i) and $\lambda /4$ (ii) thickness and dielectric mirror beginning with the higher index for the same number of layers (iii). (**f**) Corresponding reflectance spectra showing similar efficiencies for structures (i) and (iii) in reflecting light in the red part of the spectrum.

The best solution of our optimization scheme is consistently a stack of alternating layers with RI of 1.7 and 1.4, respectively, with a thickness of a quarter of the wavelength and beginning with the higher refractive index—which corresponds exactly to the description of a Bragg mirror^27^. Our results are presented Fig. 1a with a reflectance maximum at 530 nm, resulting in a strong green-colored reflection, and in Fig. 1e,iii for a maximum reflectance at 700 nm, in the red part of the spectrum. The only difference between the two is the size of the layers, the red reflecting Bragg mirror presenting larger layers because the reflected wavelength is larger - as observed in the natural example.

It is virtually impossible to assess whether an optimization has found the actual optimal solution to our problem (the best possible solution for reflecting light at a given wavelength). However, here, the fact that Bragg mirrors are systematically produced by the algorithms up to a certain number of layers strongly suggests this is the case. In any case, this result shows that the optical constraint of reflecting light efficiently alone explains the emergence of a regular pattern—which hints as to why dielectric mirrors are so ubiquitous in nature.

### Better understanding natural designs

When carried out with a higher number (up to 40) of layers, the optimization scheme produces more chaotic structures (see Supplementary Information), still systematically indicative of the fact that the RI should only alternate between 1.4 and 1.7, the most extreme values allowed—and start with the higher index facing the outermost in vacuo layer. We then force the algorithms to use these extreme values of the refractive index. When the structure begins with the higher index, perfect Bragg mirrors are retrieved beyond 40 layers (see Supplementary Information). But when the structure begins with a lower RI, a slightly different dielectric mirror emerges, with a first layer twice as thick ($\lambda /2$) as expected for a Bragg mirror.

A physical analysis allows to understand the functional role of this layer. A lower RI layer with quarter-wave thickness and a RI of 1.4 in fact constitutes an anti-reflective coating—thus lowering the reflectance. A first layer of half a wavelength constitutes actually an “absent layer”^27^ allowing to obtain the performances of a standard dielectric mirror while beginning with a lower refractive index. Man-made dielectric mirrors always begin with the higher RI medium for the above reasons^27^. However, existing literature shows that part of the elytral cuticles of the Japanese jewel beetle, *Chrysochroa fulgidissima* (see Fig. 1), are actually covered with multilayers following this exact design principle^29–31^. For a low number of layers, as is the case of the purple stripes of *Chrysochroa fulgidissima*, the reflectance can actually be doubled with this recipe (see Fig. 1). This indicates that beginning with a low index layer is a supplementary constraint and that the design we have found by optimization is the solution that has emerged spontaneously in this species as a response. The constraint may be linked to the *in vivo* development of these structures and the way the melanin layers are expressed.

This demonstrates that numerical optimization of photonic structures is able to point out specific features of natural structures that went essentially unnoticed^31^ as well as to retrieve perfectly regular structures.

**Figure 2:**
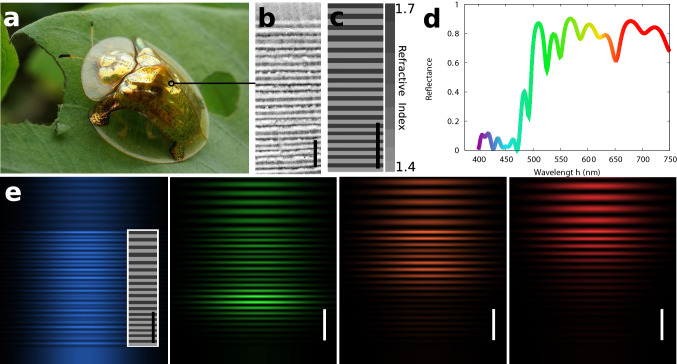
Retrieving chirped dielectric mirrors. (**a**) *Aspidomorpha tecta*, “Fool’s gold beetle”, photograph by Indri Basuki. (**b**) TEM image of the structure on the cuticule, taken from^32^. (**c**) Result of the optimization by evolutionary algorithms with a larger period at the top than at the bottom. (**d**) Reflection spectrum of the structure. (**e**) Electric field distribution map upon normal-incidence illumination, obtained using Moosh^25^, showing how different wavelength (and thus colors) are reflected (or not) at different depth in the chirped dielectric mirror. From left to right: blue (400 nm), green (530 nm), orange (600 nm) and red (700 nm). In the blue region of the spectrum (left), light can be seen crossing the whole structure without any damping and propagating again in the substrate. Scale bars: 1 μm.

### Retrieving chirped dielectric mirrors

As shown in Fig. 1, periodic dielectric mirrors possess a fixed bandwidth, determined by the RI contrast and the number of layers. Trying to tackle a more complex problem for which solutions exist both in nature and in technology, we subsequently changed the objective function to optimise a broadband dielectric mirror, i.e. a multilayered structure that reflects several wavelengths in an interval much larger than the bandwidth of a dielectric mirror. Practically, the algorithms are asked to maximize the average reflectance of the structure for 50 wavelengths between 500 and 800 nm, the RI of the layers being given. The computation time for one run is of the order of a few minutes (roughly 50 times longer than for the Bragg mirror).

The most favorable designs produced by the algorithms are dielectric mirrors with slowly varying thicknesses, as shown in Fig. 2, in which the different wavelength are reflected at different depths. Such devices are known as chirped dielectric mirrors, commonly found on metallic scarab beetles^33^ and butterfly pupae^34^, but also employed in broad-band optics^35^. Note that an inverted organization of the layers would have the same optical response and is proposed in approximately half of the solutions generated by the optimization (see Supplementary Information).

These results clearly show that regular but non-periodic architectures, which are much more complex than Bragg mirrors, can also be retrieved using evolutionary algorithms.

### Retrieving the Morpho wing scale architecture

**Figure 3:**
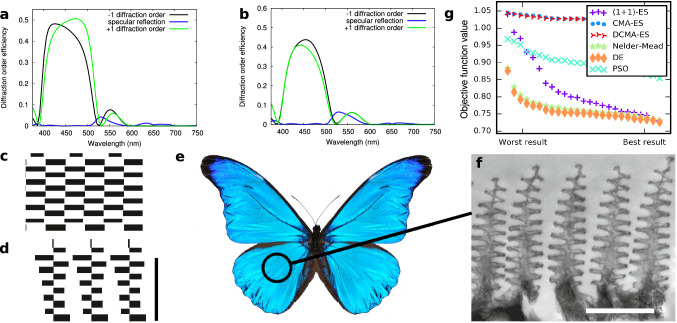
Retrieving the *Morpho* wing scale architecture.**a** Diffraction efficiency of the diffraction orders for the optimal structure (shown in **c**) found by the algorithms with no constraint except for the horizontal periodicity (fixed). **b** Diffraction efficiencies for the structure found (shown in **d**) when including a fabrication constraint and a pressure towards a lighter architecture. The bar represents 1 μm. **e** Actual view of a *Morpho rethenor*, photograph by John Nielsen. **f** TEM images of the cuticular surface of *Morpho rhetenor* (taken from^5^). The bar represents 1 μm. **g** Score (lowest value of the objective function reached) for each algorithm with 12 layers and penalization; the x-axis represents the different runs, sorted (best run on the right). See Supplementary Information.

One of the most well-known photonic structure in nature is undoubtedly the architecture that can be found in *Morpho* butterfly wing scales^3,5,6^. In *Morpho* butterflies, the wing scale ridges are folded into multilayers that resemble a Christmas-tree structure in cross-section, which is continuous along the length of the wing scales (see Fig. 3).

A multilayered structure is unable to scatter light as the incoming light is reflected by the structure following the laws of reflection, similar to the reflection on any metallic surface. Such a specular reflection can clearly be seen in the insects shown in Fig. 1 and especially Fig. 2 as bright areas. On the contrary, the structure on the wing of *Morpho* butterflies presents a horizontal periodicity, thus constituting a diffraction grating able to scatter light in different directions^36^. It is usually assumed that the role of the architecture shown Fig. 3 is to scatter blue light efficiently.

The structure to be optimized is thus constituted of rectangular blocks of cuticular chitin (RI of 1.56) with arbitrary dimensions and position, forming a periodic structure with a fixed horizontal period, a classic approach to model this kind of architecture^37^. Each block is separated from the others by an air layer of arbitrary thickness. The algorithms are allowed to modify the height, width, position of the blocks and the thickness of the air layer. They are asked to minimize the specular reflection at any given wavelength, and also to maximize the scattering of blue light (450 nm) in the higher diffraction orders, in order to reproduce the line-like scattering pattern observed in *Morpho* butterfly wing scales^6^ (see “Methods” for the detailed objective function).

Despite the jump in complexity, requiring advanced numerical methods^38,39^ that are much more costly (one run of an optimization may take up to 10 hours on a single core), the algorithms produce very efficient structures that have less than 0.0001% specular reflection whereas 98% is scattered into the diffraction orders due to an intertwined arrangement of blocks that resemble the *Morpho* wing scale nanostructures (see Fig. 3). The interdigitation is clearly responsible for the almost total cancellation of the specular reflection, indicating that the evolutionary constraint of maximizing scattering made such architectures potentially emerge.

Obviously, the actual *Morpho* structure is not optically optimal in so far as the biological construction of the structure will come with a set of other constraints that are difficult to evaluate. We have thus added additional constraints with the aim of reproducing the natural structures: (i) the blocks should be on top of each other, and (ii) the structure should be as light as possible. When the latter constraint, controlled by a parameter in the objective function (see “Methods”), is strong enough, structures that are optically only slightly sub-optimal and very close to actual *Morpho* structures clearly emerge (see Fig. 3). The structures are not as regular as the previous ones, probably because of the importance of the mechanical constraints we imposed, but the disorder which can be seen on the produced designs does not seem to have an impact on the width of the reflectance peak in the blue part of the spectrum.

These results indicate that evolutionary algorithms are not only able to yield regular, elegant and complex solutions to various optical problems, but that this process allows to understand the precise purpose of each of their features and even to quantify the balance between the optical and mechanical constraints.

## Anti-reflective coatings produced by evolutionary optimization

To demonstrate that our approach is generalizable and not limited to natural photonic structures per se, we consider the problem of an anti-reflective coating on a solar cell based on amorphous silicon illuminated in normal incidence. Such coatings have been largely studied and optimized in the past^7,27^. Here, we run an optimization for various numbers of layers (up to 20), imposing a maximum RI contrast (alternating layers of 1.4 and 1.7 index beginning with the lower index in this case), searching for the highest solar cell performance (*i. e.* conversion efficiency) for two very different thicknesses of the silicon layer (89 nm and 10 μm).

**Figure 4:**
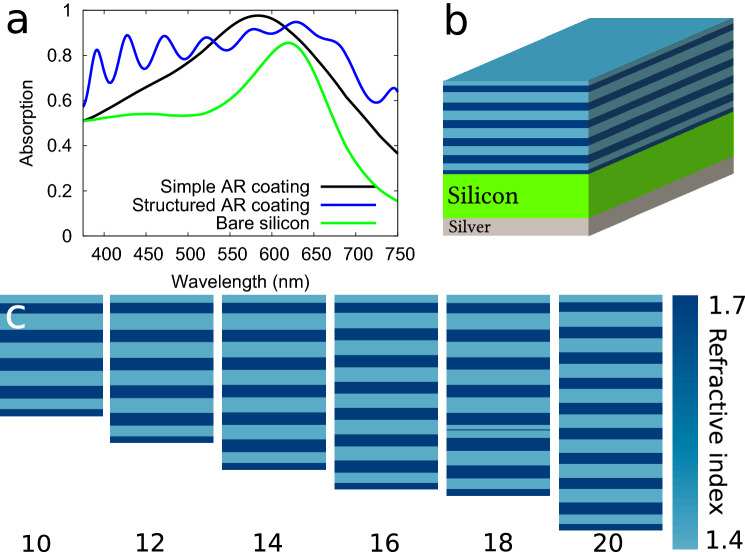
Anti-reflective coatings produced by evolutionary optimization. (**a**) Absorption spectra for a 89 nm thick amorphous silicon layer covered, bare or covered with different anti-reflective coatings. (**b**) Scheme of the structure with the multilayered anti-reflective coating designed by the algorithms with 12 layers. (**c**) Results of the optimization for different numbers of layers on top of a 10 μm thick amorphous silicon layer.

The results for both cases are shown in Fig. 4. In both cases, the produced design is clearly a modified quarterwave stack reflecting only the infra-red part of the spectrum, with the two upper and two lower layers presenting a reverse pattern, different from the dielectric mirror pattern. This result does not depend on the number of layers imposed, as shown Fig. 4. The performance of such a device is excellent, allowing to absorb about 80% of the incident visible photons inside a 89 nm thick amorphous silicon layer and clearly outperforming a standard quarterwave anti-reflective coating (see Fig. 4 and Supplementary Information). It must be stressed that the core of the structure is obviously a Bragg *mirror*—a surprising result, as this kind of structure is usually not considered as a relevant solution for an anti-reflective coating. However, a quick calculation shows that the high reflectance band of this structure is around 850 nm, in the infra-red. These multilayered coatings have thus the unique property of reflecting infra-red light very efficiently. This could potentially prevent solar cells from overheating, a phenomenon known to lower their efficiency.

Our strategy allows us to conclude, quite counter-intuitively, that slightly modified dielectric mirrors can be turned into efficient anti-reflective coatings. Photonic crystals like Bragg mirrors are usually used for reflecting light, leveraging the fact that they possess a photonic bandgap, thus forbidding light to travel inside the structure^40^. Our results suggest that their properties outside of the bandgap can be utilized outside their normal scope.

## Discussion

### Comparison of evolutionary strategies

To investigate the evolutionary optimization of photonic structures, we have applied different algorithms. Their performances significantly differed. Each algorithm was given the same budget of $10^4$ evaluations of the objective function, which is enough for each algorithm to converge towards a solution. In order to evaluate how reliable each algorithm is, we have run 100 optimizations for each algorithm (see Fig. 3). Whether multilayered or more complex structures are considered, the most widespread variant of Differential Evolution (DE) consistently outperforms the others including non-evolutionary algorithms, as shown on Fig. 3 (see Supplementary Information for the complete results). This algorithm is the only one that is inspired by sexual evolution, where recombination (by crossover rather than by averaging) between individuals plays a central role. At each generation, an individual generates an offspring that replaces its parent only if it is “fitter”. A characteristic of a parent (like the thickness of a given layer, or the width of a block) has one in two chances to be transferred directly to the offspring. Otherwise, this characteristic will take a value computed by mixing the characteristics of four individuals: the parent, two randomly chosen individuals and the best individual so far (see Supplementary Information).

In more classical numerical problems used to compare optimization algorithms, DE does not necessarily fare better—we attribute this discrepancy to the fact that the physical problems considered here are modular: each part of the structure, even if it interacts with the rest of the architecture, has a precise purpose that can be optimized partially independently. DE presents features that help in such a situation, namely combining exact copies of some variables and new variables from other individuals.

### Limitations

We first discuss here what the typical dimension of a photonic problem should be to be tackled with our method (from 5 to 40 dimension typically).

Our results do not imply that DE is the best choice for an arbitrary photonic problem. To perform well, the problem and its most efficient solutions have to be modular. If it makes little sense to optimize a part of the structure almost independently of the rest, the advantage of DE will not play out. Recent results^41^ show that for some problems in photonics with a low number of dimensions, DE is in fact among the worst possible choices. We insist that DE is more relevant for modular design problems, which implies a relatively large number of parameters (more than 5 typically). Obviously, as exemplified above, a large number of problems in photonics are modular, but not all design problems may be^7^.

Optimization problems in photonics are generally difficult as the structures are resonant. In an optical filter, for example, each layer may resonate for various frequencies, and such resonances may enhance transmission or reflection for a given wavelength. As a result, even a relatively simple photonics problem presents a number of local minima for the objective function, i.e. solutions which are locally optimal and which would require a large change to be improved further. This issue is difficult to tackle even for a global optimization algorithm and almost impossible for a local optimization algorithm^42^. As the dimension of the problem increases, the likelihood for a global algorithm to get stuck in a local minimum increases significantly: in this work, above 40 parameters, the algorithms may not be very reliable, even for DE in the most modular cases.

Finally, since there is no way to be sure a solution produced by optimization is truly optimal^42^, these have to be taken with care. One in particularly always has to question whether the solutions are regular because they *are* optimal or whether the are produced because regular solutions are easier to find. We note however that all the regular structures we have generated in this work proved to be the best solutions that could be generated in a very consistent way. Moreover, very similar solutions were produced when we gradually increased the complexity of each problem—which is a way to reinforce the confidence one can have in the generated designs. As is shown in more detail in the Supplementary Information, for a given number of degrees of freedom either the algorithms provided a regular solution which could not be beaten by any disordered structure, or no regular solution was produced at all and the algorithms ceased to be reliable (thus producing structures with a higher objective function than for a smaller number of dimensions for instance). It has been noted in a previous work^19^ that “periodicity is a principal condition for effective control of the distribution of light”. We strongly agree and hypothesize that in photonics, regularity should be considered a sign that a structure is close to optimality. Many disordered structures have probably emerged in previous works^16^ because the problem was simply too difficult for the algorithms.

### Comparison to other inverse design methods

We underline that many other problems in the optical science and related fields could benefit from such an evolutionary optimisation approach, all the more so that DE is a very simple algorithm that requires only a few lines of code. We have, for example, compared DE to the Needle algorithm for the design of optical filters^7,8^. This algorithm produces extremely efficient optical filters by adding layers to the structure to improve their performance. No other algorithm has produced better designs so reliably—thus making Needle a reference technique even though the designs are known not to be optimal^9^. In the test case of the high reflectance dielectric mirror resembling the dielectric mirror above^7^, DE was able to produce a better design (see Supplementary Information) suggesting it may compete with Needle on the most modular problems as long as the number of layer is kept low enough for DE to provide satisfactory solutions (typically under 40, see above).

Recently, topological optimization has attracted a lot of interest in the photonics community^15^. The general idea is to consider a problem with very little constraints on the geometry and thus a very large number of parameters. One then improves gradually the structure because the gradient of the objective function (and thus the general direction in which the performances improve) can be very easily computed in the case of a photonic problem^15^. Such a technique has proven extremely successful in mechanics, to the point that global optimization methods appear useless^43^. The structures produced by the optimization are regular and understandable. In photonics, because of the numerous local minima due to optical resonances (see “Discussion” above), the technique has not produced results that are similarly convincing. Generally, topological optimization generates regular structures only when periodicity or symmetry is assumed and when the starting point is already periodical (like a photonic crystal or gratings^44^). Often, some kind of regularization is performed to avoid the apparition of extremely small features. Some works use several of these techniques combined to obtain efficient and regular designs^45^. Without such steps, the methods do generate quite high-performing, but very often disordered, results that include extremely small features—thus requiring fabrication techniques which makes their commercialization unlikely^15^. This can be understood because topological optimization is inherently a steepest descent which is more likely to be stuck in one of the numerous local minima of the problem, something which does not happen in mechanics^43^. However, even if a physical analysis may lead to the conclusion that the design is not fully optimal^46^, the structures produced present excellent performance^11,12^. We underline that the choice of the starting point for the algorithm is always a delicate question^11^.

It may be difficult or even impossible to compare a global approach to a topological optimization approach. Our approach, since it does not produce satisfactory results for a very high number of dimensions, requires additional constraints on the geometry. In the case of the *Morpho* butterfly structure, we assumed a geometry composed as blocks and were able to retrieve multilayered structures (see Fig. 3). For this, we needed to limit the number of degrees of freedom for the algorithms and thus potentially introduced some bias. It remains to be seen if our global approach, which requires a lot of evaluations of the objective function, will be able to generate 3D structures with comparable performances, when more degrees of freedom will be needed. One could however imagine to combine these approaches in the future to benefit from their respective strengths.

## Conclusion

By showing that the various biological photonic structures that we have investigated are indeed optimized solutions to different well posed problems, this study provides insights regarding nature’s rationale for the observed structures—and strengthen at the same time the idea that evolution itself is an efficient optimization process. Our optimization approach has produced very regular solutions in a systematic way starting from random structures. This implies that the regularity of biological structures likely results from optical constraints.

Improving optimization algorithms requires complex problems for which the best solution is known. This is exceedingly rare, as determining that a given solution is actually optimal can be extremely difficult, if not impossible^42^. This work shows that nature has provided us with a whole class of test-bed problems *and their solution*, which will be extremely useful to design future optimization algorithms. That is the reason why the codes which have been used to produce the above results are readily available by downloading the Nevergrad library^47^.

In all our cases, the most successful evolutionary algorithm is the only one where individuals explicitly exchange information. Although its operators have been designed independently of the present work, Differential Evolution seems to be a *pot-pourri* of the strategies of sexual evolution—selection of the fittest, gene crossover and mixing of up to four genomes, role of the best individual in the reproduction process. By showing that these very features make the optimization process more efficient and allow to find the most complex and elegant architectures that occur in nature, our study suggests that sexual reproduction might bring an evolutionary advantage, which is still debated in biology^48,49^.

## Methods

We use the following evolutionary and non-evolutionary optimization methods: Nelder–Mead (NM^20^), Particle Swarm Optimization (PSO^22^), Differential Evolution (DE^21^), Covariance Matrix Adaptation (CMA^23^), One-plus-one Evolution Strategy(OPO^24^). NM (a.k.a. simplex) is not evolutionary; it is based on averaging and symmetrizing, and is considered as a fast mathematical programming solution for unreliable gradients. CMA is “half” evolutionary; it uses selection of the best, but also statistics on the global population for guiding mutations; it outperforms most mathematical programming methods and evolutionary algorithms on a wide range of artificial testbeds^50,51^. PSO adds non-biological inertial forces to evolution, leading to improved rates on at least partially separable functions. OPO is evolutionary, very simple, with blind mutations, but no recombination; it works quite well on simple problems with good conditioning. DE is the most evolutionary of these methods for various criteria: it has recombination (contrarily to OPO), this recombination is coordinate-wise so that we can modify different parts of the genome selectively (only tested algorithm with this property), and recombination a limited number of parents (contrarily to CMA). DE is recommended for real world problems involving modularity^52^, which matches our setting and biological settings.

The objective function are evaluated using either a freely available scattering matrix method^25^ for the computation of the optical properties of multilayers (reflection or absorption and short-circuit current for photovoltaic structures). In that case, we simply note $r(\lambda )$ the reflection coefficient at a wavelength $\lambda$. For the Morpho structures we use a Fourier Modal Method^38,39^ to compute the power distribution in the different diffraction orders. We note $r_i(\lambda )$ the amplitude of the *i*-th diffraction order in reflection at a wavelength $\lambda$.

The objective functions are (i) $1-r(\lambda _0)$ where $\lambda _0$ is the working wavelength to retrieve the Bragg mirror (ii) $1-\frac{1}{N}\sum _{n=0}^7 r(500+50\times n)$ (i.e. one minus the mean reflection coefficient for fifty equidistant values of the wavelength from 500 nm to 800 nm) to retrieve the chirped dielectric mirror (iii) $1-\frac{1}{2}(r_{+1}(450)+r_{-1}(450)-r_0(450))+\frac{1}{N}\sum _{i=1}^N r_0(\lambda _i)+\frac{a}{n_{b}}\sum _{j=1}^{n_{b}} \frac{w_j}{d}$ to retrieve Morpho-like structures, where $n_b$ is the number of blocks allowed, *a* is the constraint put on the weight of the structure ($a=0$ to get the optical optimum, $a=0.5$ to retrieve architectures similar to Morpho and $w_j$ the width of the *j*-th block) and the $\lambda _i$ are $N=8$ wavelength evenly distributed in the spectrum (iv) $1-\eta$ where $\eta$ is the conversion efficiency for the photovoltaic device defined as the ratio of the short-current circuit (assuming a quantum yield of one and a AM1.5 solar spectrum) to the maximum short-circuit current achievable for the spectral range considered (from 375 to 750 nm).

We underline that the codes we have used, including the Fourier Modal Method, are now part of the open Nevergrad library^47^, so that they can be downloaded freely.

## Supplementary information

Supplementary Information 1.
