# Supplementary material for: Evolutionary algorithms converge towards evolved biological photonic structures
Source: Sci Rep. 2020 Jul 21;10:12024. doi: 10.1038/s41598-020-68719-3 (PMC7374560; doi:10.1038/s41598-020-68719-3)
Supplement: Supplementary file 1 — Supplementary Information 1. [file 41598_2020_68719_MOESM1_ESM.pdf]

Supplementary Information: Evolutionary algorithms converge towards evolved biological photonic structures

Mamadou Aliou Barry,[1] Vincent Berthier,[2] Bodo D. Wilts,[3] Marie-Claire Cambourieux,[1] Pauline Bennet,[1] Rémi Pollès,[1] Olivier Teytaud,[2,4] Emmanuel Centeno,[1] Nicolas Biais,[5] and Antoine Moreau*[1]

[1]*Université Clermont Auvergne, CNRS, Institut Pascal, 63000 Clermont-Ferrand, France*

[2]*TAO, Inria, LRI, Université Paris-Saclay CNRS UMR 6823*

[3]*Adolphe Merkle Institute, University of Fribourg,*

Chemin des Verdiers 4, 1700 Fribourg, Switzerland

[4]*Facebook AI Research, 6 rue Menars, 75000 Paris, France*

[5]*Graduate Center of CUNY and Department of Biology,*

CUNY Brooklyn College, NY 11210 New York, U.S.A.

I. PHOTONIC STRUCTURES AND EVOLUTIONARY OPTIMIZATION

In the article, we state that "despite the apparent simplicity of the problem, no computational optimization algorithm has ever yielded Bragg mirrors as a solution, not even early attempts with evolutionary algorithms". We would like to develop this point here in more details.

Since the mid 90's, several groups have attempted to use evolutionary algorithms to optimize complex photonic structures, mainly focusing on multilayered structures because their properties are relatively easy to compute[19,34,39,48,49]. The results produced by the algorithms were constantly disordered or "aperiodic". As a consequence, the architectures were definitively not physically intelligible and thus did not attract much attention. Ten years ago, however, a work seemed to show that regularity could be made to appear spontaneously[16], with very little follow-up work.

We believe that two main reasons explain frequent failures at retrieving regular structures:

- The algorithms have improved a lot in the last two decades because the different techniques and operators (recombination, mutation) have been thoroughly tested and compared on well defined test-cases. The original Genetic Algorithms, while definitely being inspired by natural evolutionary strategies, relied on unnecessary ingredients (binary coding to mimic the DNA) making them less relevant and requiring costly checks on the validity of the solutions generated. This made them less efficient[25] despite some clear successes[3]. Specifically designed algorithms also suffer from many drawbacks because they have not been tested on well known cases[49]. Evolutionary strategies have since then been proven more efficient, even on the early successes treated using GA[44]. One of the leading evolutionary algorithms up to date is Differential Evolution[41] (see below). It is the heir of Genetic Algorithms, in the sense that it shares some of their philosophy (in particular crossovers which selects genes from the a parent and newly generated genes) but without using bit-representation of floating-point numbers as in the early times.

- The objective functions that were used may have complicated the task of the algorithms because they were chosen arbitrarily and were hence likely to be not well adapted to the specific problem at hand[34]. We stress that it is remarkable that the objective functions we use in the article are among the simplest that can be imagined. We have often tried several objective functions to get closer to natural structures – in each case, the simplest objective function gave the 'best' results.

Since these early attempts, genetic or evolutionary algorithms have rather been (successfully) used by members of the photonics community for problems presenting few degrees of freedom and a reduced

search space. In such cases however, no periodicity could emerge due to the large number of degrees of freedom required to see periodicity appear.

Our work suggests that there is no need to design specific algorithms for photonic structures, but that only well known algorithms (like e.g. DE), that have been extensively tested on other kind of problems have a chance to yield satisfactory results.

We underline that once an algorithm produces a solution, it is impossible to assess whether this solution is optimal or not. In the case of photonic structures however, solutions that are not intelligible and do not show any kind of regularity should be considered suspicious, as our results below indicate. The lack of any kind of regularity is a clue that the solution is probably not fully optimal.

II. OPTIMIZATION AND MODULARITY

There are many works done to test optimization algorithms, most of them on artificial benchmarks[21,42]. While there are fewer work focused on benchmarks rooted in real world problems, some studies exist[4,15,17]. Looking further than simple tests of the algorithms, we find many applications in a wide range of domains: physics, economics, biology, imagery, etc.

One of the many applications of stochastic optimization in biology[1,2,8] is to identify and test models that are best able to fit the real world. This can also serve to validate - or invalidate - hypotheses about a given biological phenomenon. Here, we focus on one such phenomenon, regarding the properties exhibited by the cuticles of beetles or the wings of butterflies to reflect light with a much better efficiency than most artificial dyes and structures can. Our hypothesis is that this problem is modular, and that this has an impact on which algorithms can tackle it effectively. We first compare optimizers in terms of performance on our test problems, and then discuss the specificities of our test case which might explain our results.

A. Optimization algorithms

The optimization problems that we consider have the following key characteristics: i) a gradient is expensive and ii) the objective function is not convex. We therefore chose five (plus one variant) of the most used optimizers in the literature in such a context:

1. $(1+1)-ES$, with a step-size updated according to the one-fifth rule[38];

2. **Covariance Matrix Adaptation Evolution Strategy (CMAES[24])**, an algorithm based on the adaptation of a covariance matrix used for generating new candidate solutions, where cumulative step-size adaptation drives the mutation rate. Also, a variant of CMAES was used, where

instead of drawing the mutations as a classical random Gaussian (independent, identically distributed) $\mathcal{N}(0, 1)$, they were generated as a quasi-random Gaussian sample[43]. We point out that quasi-random samples are low discrepancy sequences, so that this variant ensures a better search over each axis and each combination of axes - hence, combinations of mutations are more likely to be evenly tested.

3. **Nelder-Mead[36] (NM)** (also known as the amoeba method), a simplex method where only one point is moved at each generation, unless no better point could be generated, in which case almost all points are moved.

4. **Differential Evolution[41] (DE)**, with the DE/currToBest/1 variant. Each mutation is (in the usual version considered here) the product of a crossover between three individuals and the best one in the current generation.

5. **Particle Swarm Optimization[28,40]**, an algorithm where individuals evolve according to their own velocity, which is influenced by positions of known good individuals.

As DE will be central in the present work, we include a more precise definition: there is a population; at each generation, each 'member' x in the population generates an offspring; this offspring is a binary crossover between x and some mutation $y(x)$ of x. $y(x)$ is obtained through mutations. In DE mutations are extracted from differences between individuals. A key formula in DE defines how $y(x)$ is defined (Table II). There are several variants and we use DE/currToBest/1. Then a binary crossover combines x and $y(x)$ into an offspring $z(x)$. This binary crossover works as follows, for a problem with d variables:

- A randomly drawn variable CR in $\{1, 2, \ldots, d\}$ accepts y, i.e. $z(x)_{CR} = y(x)_{CR}$; this ensures that $z(x)$ contains at least one variable from $y(x)$.

- For each other variable $i \neq CR$, independently, we randomly select x_i or $y(x)_i$, equally likely, for $z(x)_i$.

This offspring $z(x)$ replaces x in the next generation if and only if the objective function is better at $z(x)$ than at x. The complete pseudo-code is presented in Alg. I.

NM (a.k.a. simplex) is not evolutionary; it is based on averaging, symmetrizing or expanding a set of candidate solutions - it is considered as a fast mathematical programming solution for unreliable gradients[11]. CMAES (pseudocode in Table III) uses selection of the best, but also statistics on the global population for guiding mutations, and no crossover (in the sense: no crossover which can take exactly as a value of one variable (i.e. an allele) the value from one of the parents); and the population is reduced

to a single individual by averaging before switching to the next iteration; it outperforms many mathematical programming methods and evolutionary algorithms on a wide range of artificial non-modular testbeds[20,22]. PSO (pseudocode displayed in Table IV) adds non-biological inertial forces to evolution, leading to improved rates on at least partially separable functions. The one-plus-one evolution strategy ($(1 + 1)$-ES, Table V) is evolutionary, very simple, with blind mutations, but no crossover (not even averaging of points); it works quite well on simple problems with good conditioning.

DE is the most evolutionary of these methods for various criteria: it has recombination (contrarily to $(1 + 1)$-ES), this recombination is coordinate-wise so that we can modify different parts of the genome selectively (only tested algorithm with this property), and recombination involves a limited number of parents. DE is recommended for real world problems involving complex structures[12], which matches our setting and, in general, biological settings.

For those optimizers, only defaults or classical parameters were used: we did not attempt to tune each of them. In a work such as this one, that physicists or biologists would be interested in replicating, the ease of use of those algorithms is probably at least as important as the performances themselves. Those parameters were:

- $(1 + 1)$-ES: the step size is multiplied by $r = 1.5$ in case of a successful mutation, and divided by $r = 1.5^{1/4}$ on a failure.
- CMAES: population size $\lambda = 4 + \lfloor 3\log(N) \rfloor$, parent population size $\mu = \lambda/2$.
- NM: Population is, by design, $\mu = N + 1$, and mutation parameters are $\alpha = 1$, $\gamma = 2$, $\rho = -0.5$ and $\sigma = 0.5$.
- DE: The DE/curr-to-best/1 variant was used (mutate the selected individual with the best one of the generation) with mutation parameters $f_1 = 0.8$, $f_2 = 0.8$ and $cr = 0.5$.
- For PSO, the standard parameters are widely discussed ([6,9,10,37,46,50]): We used a population $\mu = 30$, with 10 neighbors and update parameters $\omega = \frac{1}{2 \times log(2)}$, $\Phi_p = 0.5 + log(2)$, $\Phi_g = 0.5 + log(2)$, $velocity_{init} = 1.0$ and $velocity_{max} = 1.5$

In addition, we note the different origins of those algorithms: two of them - CMAES and NM - come more or less from applied mathematics, though CMAES uses selection as other evolution strategies. The three others are more biology-related: $(1 + 1) - ES$ can be seen as an asexual reproduction; DE is akin to evolution through reproduction (albeit with more than two partners); finally PSO mimics the flight of birds, or shoals of fish, but is not related to evolution itself.

All optimizers ran ten times for any given number of layers on each of the problems for multilayers. In the case of the *Morpho* wing scale structures, the optimizers ran 100 times (for clarity of the figures we display only one run out of 4, after sorting by performance). In every instance, the maximum budget was set at 10^4 evaluations of the objective function. For solving the quarter-wave stack and the chirped dielectric mirror, the dimension of the problem was once or twice the number of layers of the mirror (depending on whether the refractive index contrast is imposed or not). The last problem, reproducing *Morpho* wing scale structures, is more complex: the overall dimension is then equal to four times the number of blocks.

B. Comparing performances

The optimizer that worked best is Differential Evolution (Fig. 3g), an optimizer inspired by sexual reproduction and natural evolution. While optimizers like CMAES or NM performed well at times, they were never able to really challenge DE. CMAES performed well on the simplest problem (common Bragg stack), not too badly on chirped structures, but got poor results on the challenging task of recovering the *Morpho* wing scale structures. The performances of the Quasi-Random variant however were in each and every case better than the vanilla version on the first and second problems. On the last one, even if it was not the case for each computed statistic, it was always the case for the best run. In fact, where the vanilla version did not reach the optimum a single time, the QR version got it in all but one case (the last one, with 12 layers), and even then, it was not far. $(1+1)-ES$ and PSO are a bit in the middle: they were not able to perform as well as DE, but still got good results for the most part.

C. Modularity

Complex real-world structures often have some kind of modularity. Modularity can be defined in various ways; we here focus on strong links inside groups of variables, at various levels of a hierarchy. We have many layers, defined by several variables each, and not all these layers interact equally strongly. Therefore it makes sense to have an optimization algorithm that takes this structure into account - namely, algorithms with crossovers which select variables randomly in one or another of the parents rather than considering a middle point between the parents.

In all our runs, whether multilayered or more complex structures are considered, DE, the only algorithm based on gene-mixing sexual reproduction, has outperformed the other algorithms. In more classical numerical problems used to compare optimization algorithms, in particular those in which modularity is

absent, these algorithms do not necessarily fare better. One way to explain this success of algorithms using gene-mixing sexual reproduction is that real world problems, including biological problems such as the present ones, do not mix all variables by artificial rotational invariance[23] and have a naturally modular structure - therefore, methods combining modules perform best, as in topology optimization[26] (not so many algorithms can optimize the shape if a chair in a non-parametric way!), control[27], planning[5], where they routinely win competitions. One of the claims of the present work is that real world problems, at least those considered here, contain a form of modularity which is

- neither (almost) full separability, for which PSO typically performs best[22];
- neither (almost) full rotation i.e. no modularity at all, for which CMAES typically performs best[20,22];
- fit for crossovers and in particular DE.

A colorful analogy, for the relevance of gene-mixing crossovers, is that a giraffe and a horse both make sense, so that we can pick up either the gene “long neck” or the gene “short neck”, but an intermediate neck is pointless - and averaging reduces the diversity to such an intermediate point. In addition, at the level of crossovers, it makes sense to check random combinations of several genes, rather than adding a random noise on a middle point of all genes - this is not only a chemical constraint of DNA crossover in nature, it is also a suitable feature for modular problems.

More broadly, it is striking to see that DE in its "current to best" variant, the most successful algorithm for our problems and whose operators have been designed independently of this work aimed at mimicking biological structures, finally looks like a *potpourri* of all the strategies of sexual evolution (selection, impact of the herd leader on reproduction, and crossover). Incidentally, CMAES was clearly improved by quasi-random mutations, which are aimed at correctly searching combined mutations - a step in the direction of modularity.

III. THE QUARTER-WAVE STACK

The quarter-wave stack[33] is a structure that has been known for decades[30–32]. When we asked the evolutionary algorithms to maximize the reflection coefficient of a multilayered structures with arbitrary indices and thicknesses the results pointed towards the quarter-wave stack beginning with the higher index, exactly as explained in textbooks[33].

The objective functions were computed using a scattering matrix algorithm for the multilayered structures – the program Moosh[13]. It constitutes the fastest unconditionally stable method to compute the

Randomly draw the population x_1,...,x_{30} in the search space.
while There is time **do**
{// This is one iteration}
for each x in the population **do**
Randomly draw a, b, c and d in the population.
Compute y using one of the equations in Table II.
Randomly draw CR in $\{1, \ldots, d\}$. {// d is the dimension}
for each i in $\{1, \ldots, d\}$ **do**
if $i = CR$ or with probability $\frac{1}{2}$ **then**
$z_i \leftarrow y_i$.
else
$z_i \leftarrow x_i$.
end if
if z has better fitness than x **then**
x is replaced by z in the population.
end if
end for
end for
end while

Table I. Pseudo-code of DE.

optical properties of any multilayer (reflection coefficient, propagation in the structure, absorption by silicon and short-circuit current in that case). The code has been recently published and is freely available[13]. The objective function is $1 - r(\lambda)$to retrieve the Bragg mirror and the Bragg mirror with a thicker top layer, where $r(\lambda)$ is the energy reflection coefficient at a wavelength λ in nanometers.

The initial individuals are chosen randomly, with a thickness comprised between 10 and 300 nm, and an index (when it is not fixed) comprised between 1.4 and 1.7. The algorithms then check that each solution they come up with respects these limits.

In the paper, we present the results for a number of layers up to 12, for which no doubt is possible. The structures produced by the algorithms are exactly Bragg mirrors. When the number of layers is increased, the results are not as perfect - or at least not all the time. Since we have imposed a fixed limit to the number of objective functions that each algorithm can perform, this was expected. We underline that optimizing a structure with 40 layers means that 80 parameters are left free, making the problem extremely complex.

Up to 24 layers, almost perfect dielectric mirrors are found except for a few layers. Sometimes $\lambda/4$ layers are replaced by $3\lambda/4$ layers. Such a change can be explained because it is almost equivalent to

$$\text{DE/rand/1:} \quad y(x) = a + F_1(b - c)$$

$$\text{DE/best/1:} \quad y(x) = best + F_1(a - b)$$

$$\text{DE/randToBest/1:} \quad y(x) = c + F_1(a - b) + F_2(best - c)$$

$$\text{DE/currToBest/1:} \quad y(x) = x + F_1(a - b) + F_2(best - x)$$

$$\text{DE/rand/2:} \quad y(x) = a + F_1(a - b + c - d)$$

$$\text{DE/best/2:} \quad y(x) = best + F1(a - b + c - d)$$

where a, b, c, d are randomly drawn, distinct, in the population. We see that the mutated variant of x, namely $y(x)$, is in some cases independent of x - this is normal.

Table II. Various DE formulas.

take a $\lambda/4$ layer or one thrice as large for the reflection coefficient at the operation wavelength – and we did put a high (300 nm) limit to the thickness of the layers, clearly allowing for such layers to emerge. However, such a change makes the bandwidth narrower. In nature, it would probably not be favored. A larger bandwidth means higher chances for the structures to be found by the algorithms, because is means a larger attraction basin. That is probably the reason why thicker layers are not found for a small number of layers.

We underline (as can be seen in the article), that as soon as a few periods are present, the performances of the device are actually very good. Any increase in the number of periods is likely to be incremental. It is almost surprising that the algorithms were actually able to produce dielectric mirrors with up to 24 layers. Above this limit, though, structures that are more or less regular emerge. They still present excellent performances, as shown by the spectra, but are only periodic by parts, and do not exactly reach the performances of a pure Bragg mirror.

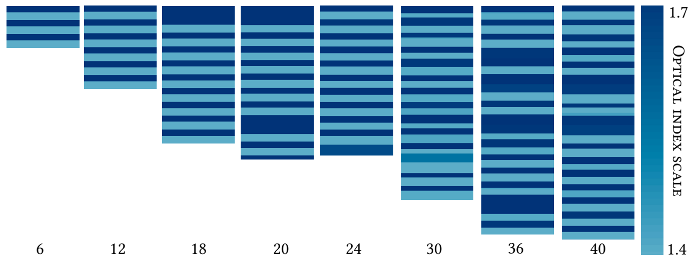

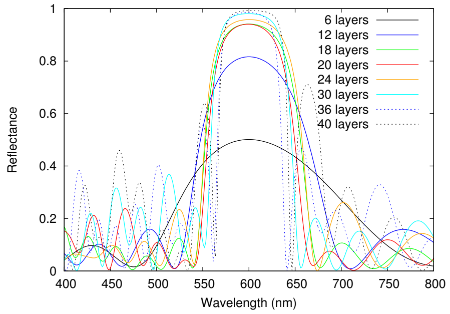

Figure 1. Results of the optimization when the algorithms are allowed to modify the optical index (in the interval $[1.4, 1.7]$) and the thicknesses of the layers. Left: the structures. Right: The corresponding reflection spectra.

Inputs: f objective function, n dimension, population size λ and parent population size $\mu < \lambda$

{Initialize:}

C =identity matrix, $p_c = 0$, $p_\sigma = 0$

$w_1, \ldots, w_\mu$ can be chosen freely, with $\mu_i \geq \mu_{i+1}$ and such that $\mu_w = 1/(\sum_{i=1}^{\mu} w_i^2) \simeq 0.3\lambda$, $c_c \simeq 4/n$, $c_\sigma \simeq c/n$, $c_1 \simeq 2/n^2$, $c_\mu \simeq \mu_w/n^2$, $c_1 + c_\mu \leq 1$, $d_\sigma = 1 + \sqrt{\mu_w/n}$

while Not terminate **do**

{Generate offspring}

For each $1 \leq i \leq \lambda$, y_i is randomly drawn, centered multivariate Gaussian with covariance C

For each $1 \leq i \leq \lambda$, $x_i \leftarrow m + \sigma y_i$

{Rank points (randomly break ties)}

Compute $f(x_i)$ for each i

Define (i) the index of the ith best offspring: $f(x_{(i)}) \leq f(x_{(i+1)})$

{Update mean}

$m \leftarrow m + \sigma z$, where $z = \sum_{i=1}^{\mu} w_i \sigma y_{(i)}$

{Cumulation for C}

$p_\sigma \leftarrow (1 - c_c) p_c$

if $p_\sigma < 1.5\sqrt{n}$ **then**

$p_c \leftarrow p_c + \sqrt{1 - (1 - c_c)^2}\sqrt{\mu_w} y_w$

end if

{Cumulation for σ}

$p_\sigma \leftarrow (1 - c_\sigma) p_\sigma + \sqrt{1 - (1 - c_\sigma)^2}\sqrt{\mu_w} C^{-\frac{1}{2}} y_w$

end while

Table III. Pseudocode of CMAES.

Despite the lack of regularity sometimes, we underlined that very few layers present, in any of the structures above, an intermediate optical index (see figure 2 for the 40 layer structure). In a layer, the index is usually either the lowest or the highest allowed (respectively 1.4 and 1.7). When this exact contrast is imposed, the problem is largely simplified for the algorithm – all the more so that it is very detrimental to the convergence to constantly bump on a constraint like the one that is imposed to the indices. In that case, the optimizations yield perfect Bragg mirrors up to 40 layers for the very least, as show Fig. 3. We have not tried to go beyond this number of layers.

Figure 4 presents a comparison between the results of the different algorithms tested on this case.

Inputs: μ population size, f objective function, initial speed norm $velocity_{init}$, maximum velocity $velocity_{max}$.

{Initialization}

for $1 \leq i \leq \mu$ **do**

$x_i \leftarrow$randomly drawn i^{th} point in the search space

$p_i \leftarrow x_i$ (best known position for particle i

$v_i \leftarrow$ randomly drawn initial speed with norm $velocity_{init}$

end for

$g =$ current best point in the population, i.e. $g = x_i$ such that $f(x_i)$ is minimal

while Not terminate **do**

for $1 \leq i \leq \mu$ **do**

for $1 \leq d \leq n$ **do**

{Let us work on coordinate d}

Randomly draw r_p and r_g uniformly in $[0, 1]$

Update $v_{i,d}$: $v_{i,d} \leftarrow \omega v_{i,d} + \phi_p r_p (p_{i,d} - x_{i,d}) + \phi_g r_g (g_d - x_{i,d})$

Clip the velocity $v_{i,d}$ to $[-velocity_{max}, velocity_{max}]$

end for

Update $x_i \leftarrow x_i + v_i$

$p_i \leftarrow best\ of(x_i, p_i)$ using f

$g \leftarrow best\ of(p_i, g)$ using f

end for

end while

Table IV. Pseudo-code of PSO.

Looking at each optimizer individual results, we can notice the overall good performances of CMAES and DE. By comparing the medians to the best individual, we can also notice that up to twenty layers, the problem seems to be quite well handled by those two optimizers: at least half of the time they were able to reach the optimal solution. While the other three optimizers managed to approach the optimal solution for ten and twenty layers, their performances then degrade to the point where for forty layers, there is a factor of two between their results and those of DE/CMAES.

IV. BEGINNING WITH THE LOWER INDEX

We have considered here the case of a dielectric mirror with fixed index, but beginning with the lower index conversely to the case above. The first layer has always a thickness that is doubled compared to the

Inputs: dimension n, objective function f, step-size σ, step-size adaptation rate r

$x \leftarrow (0, 0, \ldots, 0)$

while Not terminate **do**

  Generate $x' = x + \sigma\mathcal{N}(0, 1)$

  if $f(x') \leq f(x)$ **then**

    $x \leftarrow x'$

    $\sigma \leftarrow r\sigma$

  else

    $\sigma \leftarrow \sigma / r^{\frac{1}{4}}$

  end if

end while

Table V. Pseudo-code of the $(1 + 1)$-ES.

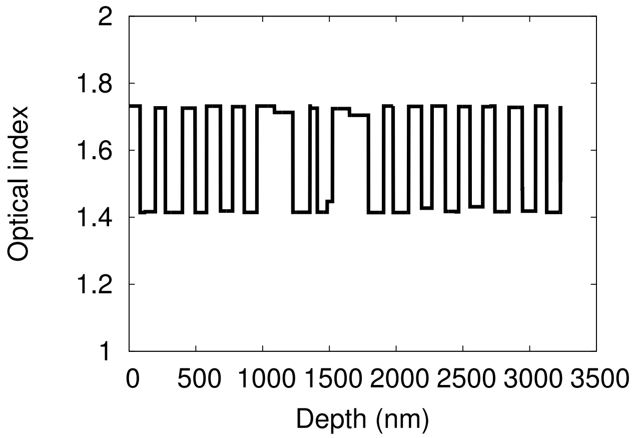

Figure 2. Optical index profile for the optimized structure presenting 40 layers. The index essentially varies abruptly from 1.4 to 1.7 and back. Almost no intermediary indices can be seen here.

other layers with the same optical index. The objective function remains the same, we have simply put a constraint on the thickness of the first layer (larger than 30 nm). Without this constraint, since they have exactly the same performances, the algorithms find half of the time the mirror beginning with the lower index and thicker layer and the standard dielectric mirror. The substrate on which the whole structure relies is assumed to be made of the same material and presents an index of 1.4. When this is not the case, although the half-wave layer improves the reflection coefficient compared to the quarter-wave layer, it is not exactly as good as when beginning with the higher index.

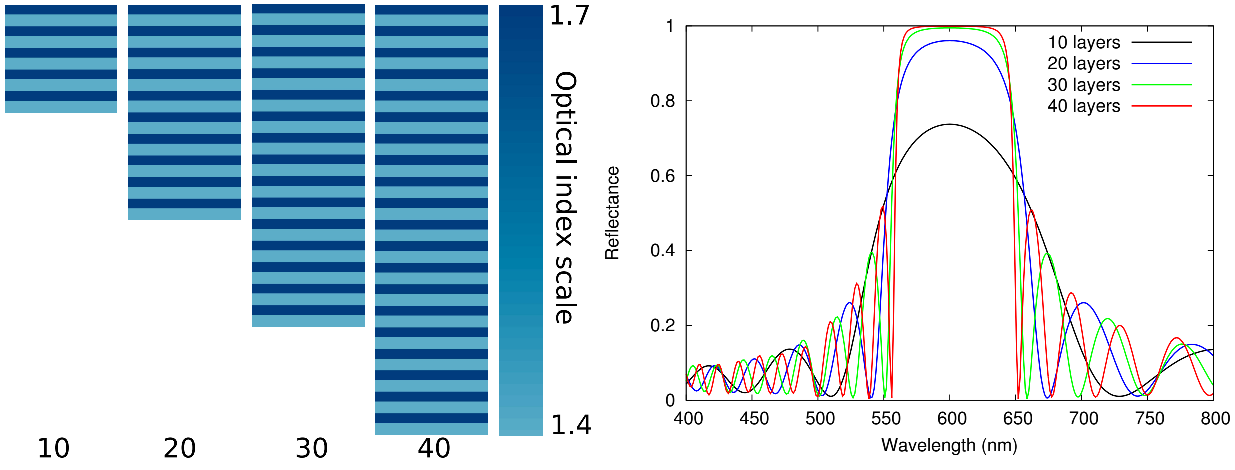

Figure 3. Results of the optimization when the algorithms use fixed optical indices, beginning with the higher index.

Layers	1 & 2	3 & 4	5 & 6	7 & 8	9 & 10	11 & 12	13 & 14	15 & 16	17 & 18	19 & 20
Odd, thin	85.148	82.220	78.148	84.214	98.724	104.391	98.556	113.842	99.507	109.016
Even, thick	103.159	97.388	98.108	109.286	144.311	118.996	132.302	125.882	125.991	127.987

Table VI. Thickness of layers in a 20 layers Chirped Mirror reflecting around 50% of the visible spectrum. Table is read top to bottom, left to right. In this mirror, a thin layer is always followed by a thicker one

V. CHIRPED DIELECTRIC MIRRORS

Looking for more broadband reflective designs than the standard quarter-wave stack, we use the objective function

$$1 - \frac{1}{8}\sum_{n=0}^{7} r(500 + 50 \times n) \tag{1}$$

where $r(\lambda)$ is the reflection coefficient in energy of the structure at the wavelength λ. This function objective is, to summarize it, one minus the mean reflection coefficient for eight equidistant values of the wavelength from 500 nm to 800 nm. We have no need to ask for some transparency in the blue part of the spectrum - which is achieved naturally for all the structures we have found. Most of the results are shown on Fig. 6 and 7. The structures not shown are totally similar. The designs resemble closely a Bragg mirror, except that the thicknesses vary slowly in the structure. Some (like for 40 layers) have a smaller period at the top, others (like for 36 layers) have a larger period at the top. Both of them present similar performances, actually. As can be seen, the larger the number of layers, the smoother the variation of the thicknesses. The thicknesses are given here for the 20 layers result in Table VI.

Looking at each optimizer's results (found on Figure 8), it is interesting to note that while the problem is a difficult one in the physics sense, it doesn't seem to be for the optimizers: up to 18 layers, all of them

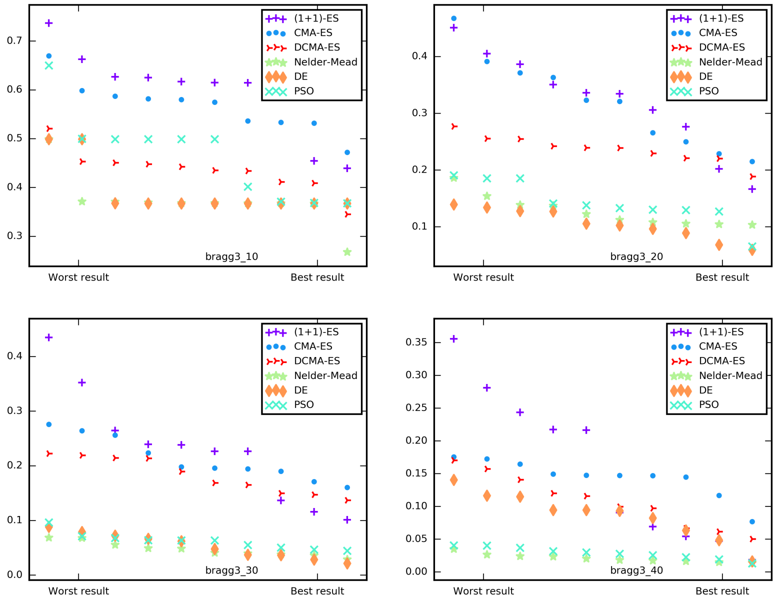

Figure 4. Optimization results for a simple Bragg Mirror. Bragg3 with 10, 20, 30 and 40 layers. With ten layers the problem seems quite simple, but in higher dimensions there are visible performance differences between the optimizers. Overall, CMAES and DE perform best, with quasi-random noticeably improving the performances of CMAES.

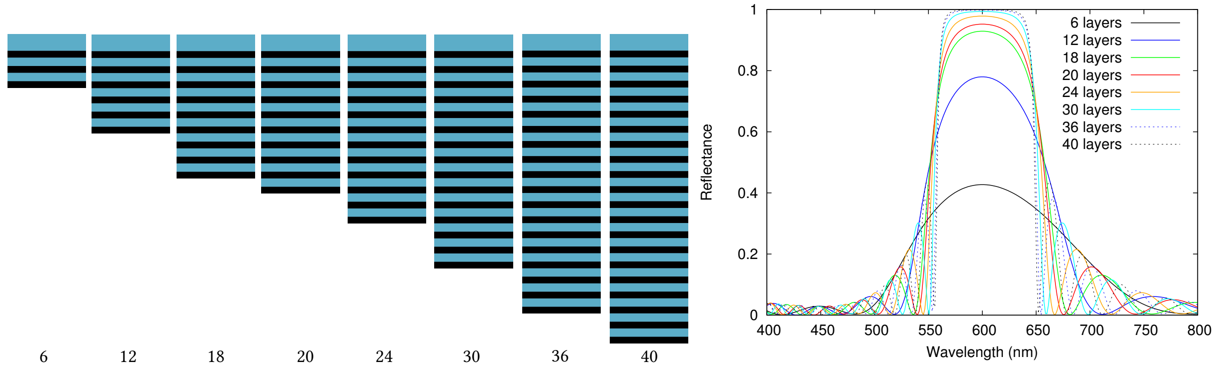

Figure 5. Results of the optimization when the algorithms when the first layer has the lower index. Left: the structures. The blue color represents a lower index layer, while the black represents a higher index layer. Right: The corresponding reflection spectra.

except PSO were able to get to the optimal solution in at least one of the ten runs. In fact, DE performed so well that it was able to find the optimum solution on almost each of its runs. While PSO wasn't able to do so, it was quite robust: on each problem, it was just a little worse than DE, and, from other statistics (mean,

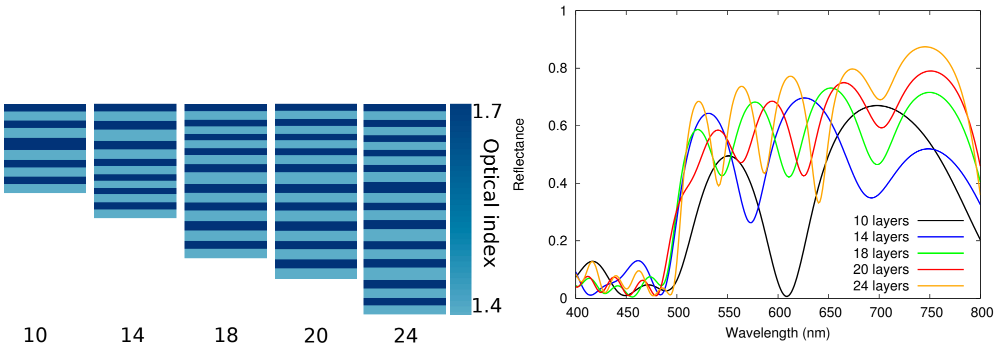

Figure 6. Left: structures produced by the algorithms, from 10 to 24 layers. Right: Corresponding reflectance spectra.

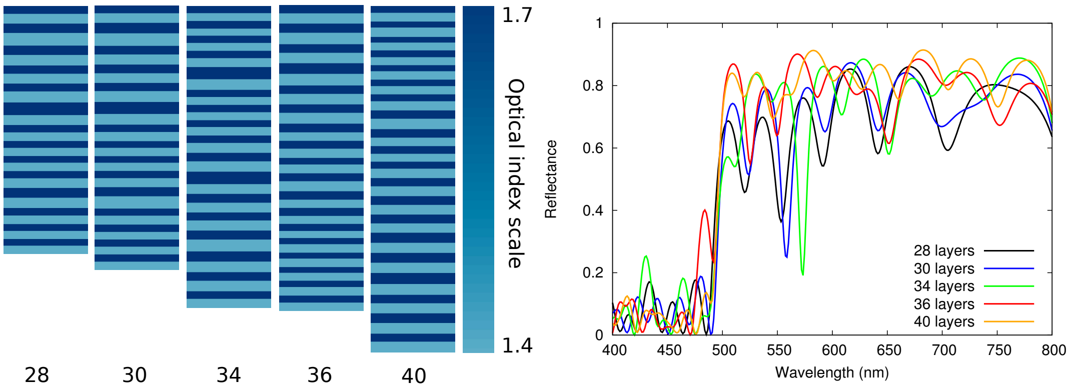

Figure 7. Left: structures produced by the algorithms, from 28 to 40 layers. Right: Corresponding reflectance spectra.

median and worst), we can see that PSO beats all the other optimizers except DE in almost all instances - though it usually fails to find the optimum.

Increasing the number of layers, we can at first glance notice that $(1+1)-ES$, CMAES and NM begin to be noticeably less consistent: $(1+1)-ES$ never gets the optimal solution for 30+ layers, the QR version of CMAES only gets it once for 32 layers, and for 20+ layers, NM only gets it for 36 and 40 layers. In the meantime, DE is once again the most consistent: reaching the optimal solution in all but 3 cases (32, 36 and 40 layers), and with better means, medians and worst results. On those indicators, PSO, NM and $(1+1)-ES$ are just behind, while both versions of CMAES are noticeably farther.

VI. RETRIEVING THE MORPHO-LIKE ARCHITECTURES

Numerical tools able to compute the optical response of a structure as complicated as the one present on Morpho butterfly wings can be extremely costly computationally. Here, we use a Fourier Modal Method[18,29] that has the advantage of being extremely fast for solving horizontally periodic problems. This method is especially suited for periodic problems, horizontally. Here we consider a period of $d = 600$

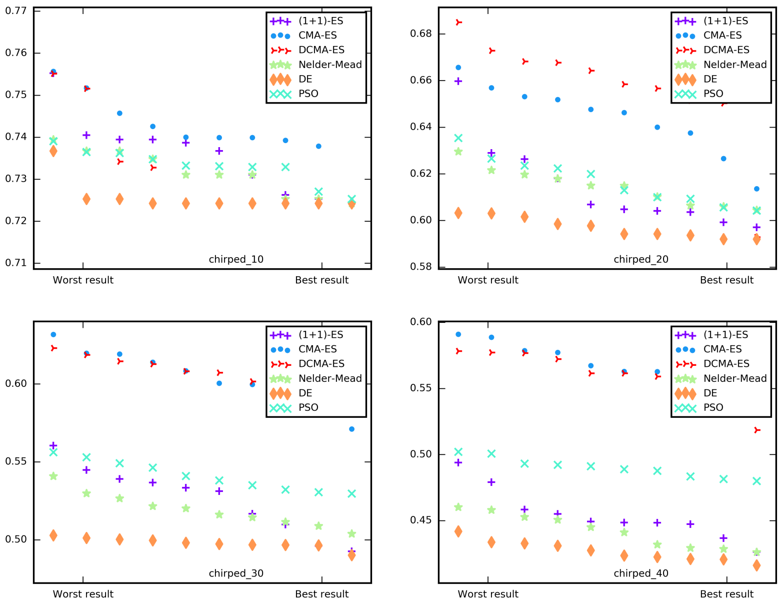

Figure 8. Chirped plots Optimization. Each point is an independent runs. Results when looking for a dielectric mirror with 6 to 40 layers, reflecting the entire visible spectrum. The x-axis corresponds to a sorting of results from worst (left) to best (right). For low dimension, no optimizer seems really better than any other. As the dimension increases however, DE really starts to outperform all the other optimizers. Only multiples of 10 layers are presented (see text for more).

nm and 25 different modes in each layer. This mode number has been chosen to get a good accuracy for a low computational cost, in order to be able to perform the optimization. The period is imposed, and we chose arbitrarily 600 nm here because this number is close to the period of actual structures[47].

The structure thus consists in a fixed number of layers, each containing one block of chitin, with an arbitrary thickness, width (smaller than d) and position within the period. For the initialization, each layer is defined by its thickness (ranging from 0 to 150 nm), its shift from the origin (from 0 to 600 nm, the full period), its width (from 0 to 600 nm) and the last is the space between this layer and the next (from 0 to 75 nm).

The modal method provides the reflection coefficients in the different orders of diffraction r_{-1}, r_0 and r_{+1} the energy reflection coefficient respectively in the -1, $0th$ and $+1$ order. The coefficient r_0 is the reflection coefficient for the specular reflection, the mirror-like reflection, obeying Snell's laws. The reflection in the diffracted orders is directly linked to the way each pattern scatters light. The $r_{\pm1}$ are not null only if $\lambda < d$ as only the specular reflection occurs for $\lambda > d$.

The objective function used to retrieve Morpho-like structures is

$$1 - \frac{1}{2}(r_{+1}(450) + r_{-1}(450) - r_0(450)) + \frac{1}{N}\sum_{i=1}^{N} r_0(\lambda_i) \tag{2}$$

with $\lambda_i = \{300, 400, 500, 600, 700, 800\}$, ensuring the maximization of the diffraction orders at 450 nm and the minimization of the specular reflection for a wide range of wavelength.

The results obtained are shown on Fig. 9 and the corresponding reflection spectra on Fig. 10.

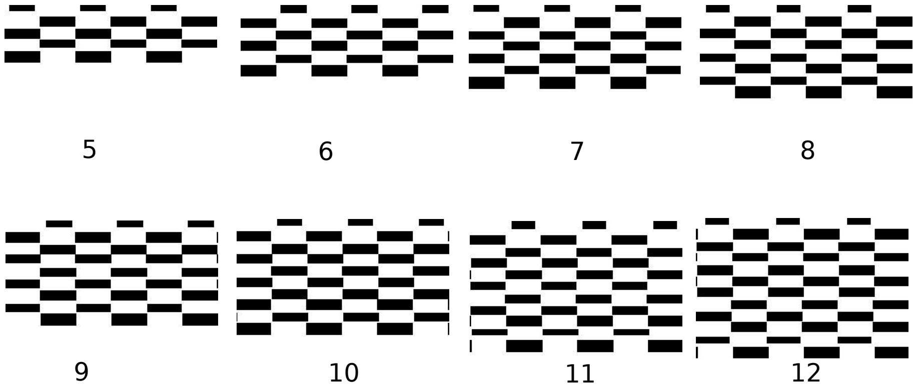

Figure 9. Structures obtained through optimization for different numbers of layers. With no constraints.

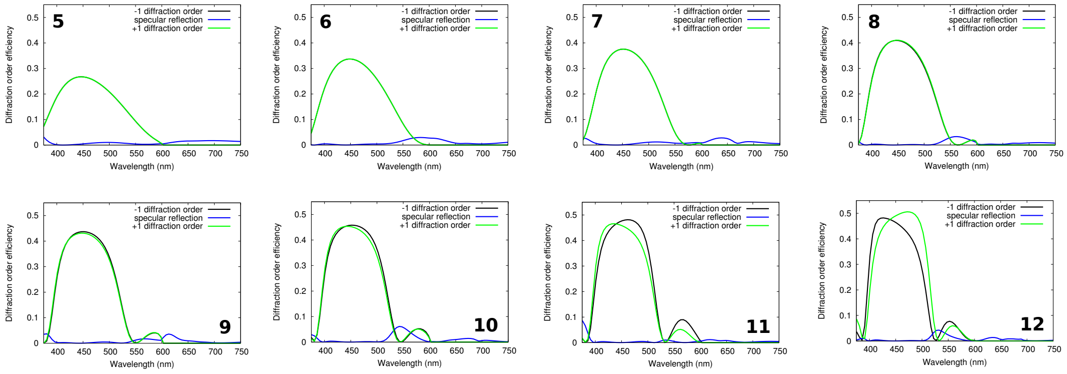

Figure 10. Spectra for structures with from 5 to 12 layers shown in figure 9.

Since other constraints, making the Morpho architecture less optically optimum, seem to play a role, we have added a term to the objective function of a times the mean filling factor of the layers (the filling factor being comprised between 0 and 1 for each layer). The objective function is now

$$1 - \frac{1}{2}(r_{+1}(450) + r_{-1}(450) - r_0(450)) + \frac{1}{N}\sum_{i=1}^{N} r_0(\lambda_i) + \frac{a}{n_b}\sum_{j=1}^{n_b}\frac{w_j}{d} \tag{3}$$

where n_b is the number of blocks assumed for the structure and w_j the width of layer j. The parameter a controls the weight of this peculiar constraint. The results shown Fig. 11 are obtained using $a = 0.5$. This

penalizes structures with a lot of matter. In the following this case is thus said to be "with penalization". As adding this sole constraint yields structures extremely similar to the first ones, but with narrower blocks, we have added a fabrication constraint: that the blocks should all be at the vertical of a chosen point. This makes architectures emerge that look very much like the actual structures on Morpho wings.

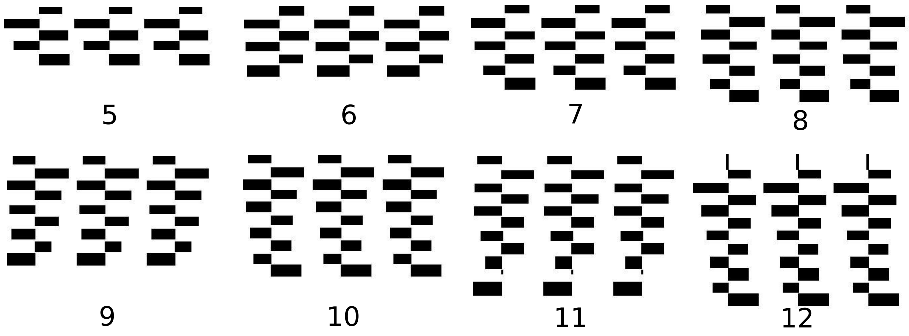

Figure 11. Structures obtained through optimization for different numbers of layers when two constraints are added: less matter and a fabrication constraint.

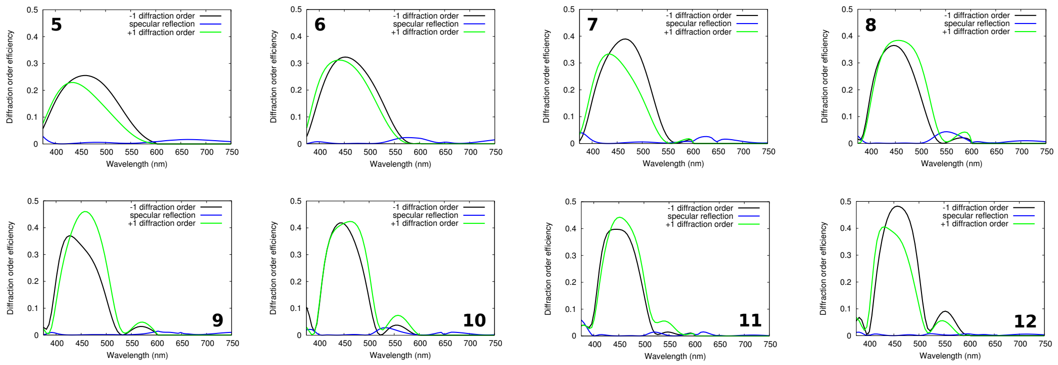

Figure 12. Spectra for structures with from 5 to 12 layers shown in figure 11.

We can safely conclude that although the optical response of the structure essentially governs the architecture, the environmental constraints at play here (fabrication constraints and the need for lighter structures) do explain the other features of the actual arrangements.

Individual results in the case without penalization are shown in Fig. 14; results without penalization in Fig. 13 show a stronger domination by DE. DE still gets excellent results, but with 11 and 12 layers is (slightly) beaten by NM, which gets very good results across the board. $(1+1)-ES$ and PSO are slightly worse, and don't perform as well on this problem than in the previous two. CMAES gets poor performances: it manages to approach the optimal with 4 and 5 layers but not quite. After that, it is quite badly outperformed by all of the others.

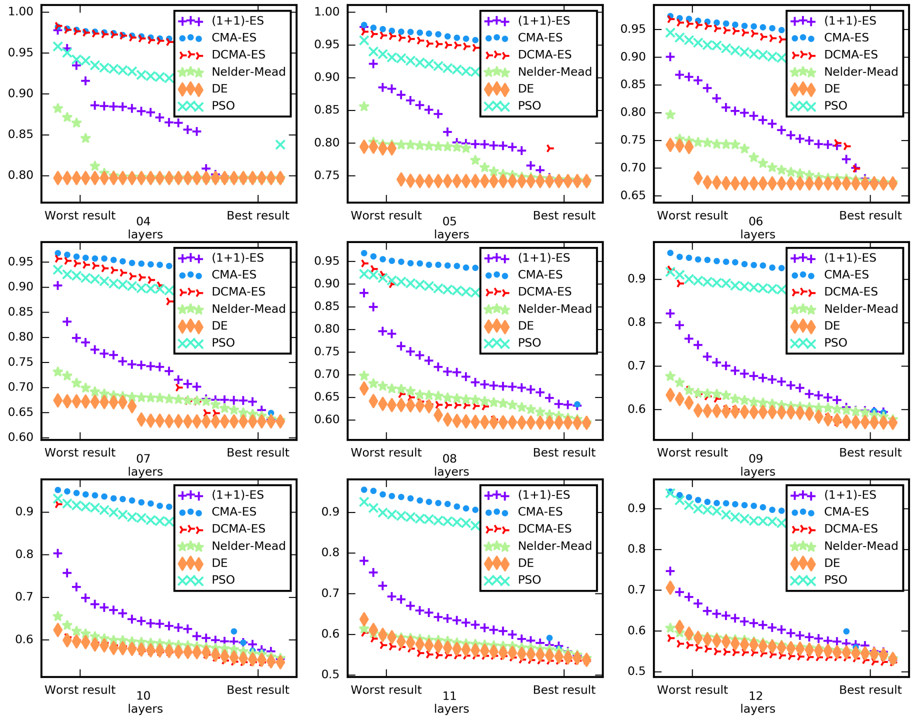

Figure 13. Performance of the different algorithms on the butterfly problem with no penalization (the lower the better). The x-axis represents the different runs (sorted: best results on the right). DE dominates for most dimensions but was less robust than NM and DCMA (i.e. rightmost results are the same but leftmost results are worse for DE).

VII. HIGH REFLECTIVE DIELECTRIC MIRRORS

The Needle technique is an algorithm which is specific to multilayers based on a discrete set of refractive index. In its original version, it adds a layer at a place it has determined will have the most impact on the performances, then chooses the optimal thickness for this layer and so on. Using this technique, it is able to reach a reflectance or a transmittance for the optical filter that can be arbitrarily close to the desired spectra. Needle is thus not strictly speaking an optimization algorithm which will generally look for a solution with a fixed number of layers - and its principle can not be extended to any other problem, especially to more complex geometries as the Morpho structure. But given how important Needle is for the community of optical filters, we had to try to compare Needle with global optimization algorithms.

Since any global optimization algorithm will begin to struggle when the number of layers becomes too large (as shown above, above 40 degrees of freedom, global optimization algorithms fail repeatedly to find the optimum), Needle is able to "beat" any global optimization algorithm. However, it is well

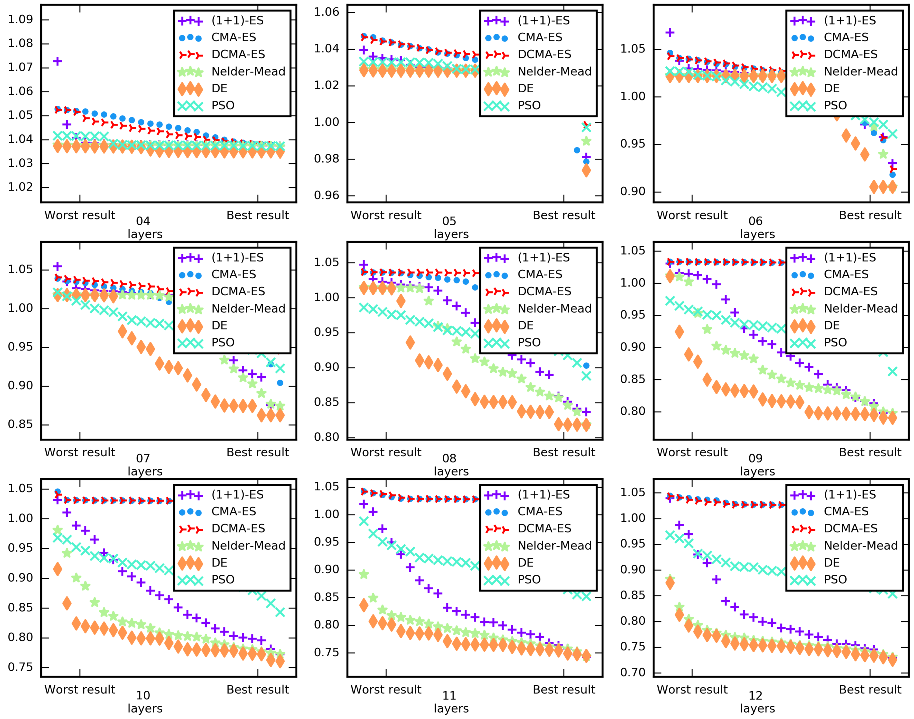

Figure 14. Performance of the different algorithms on the butterfly problem with penalization (the lower the better). The x-axis represents the different runs (sorted: best results on the right). DE dominates.

known too that the solutions provided by Needle are not optimal for a given number of layers. When the number of layers is low enough, efficient global optimization algorithms as DE may be able to provide better solutions than Needle. Again, Needle works by adding layers, so that no comparison can be fair to both kinds of algorithms. We have tried just to see if at least Differential Evolution could compete with Needle somehow, using standard test cases[45], especially the most modular one: the high reflectance filter, which looks actually like a chirped dielectric mirror. The only difference with the case above is the range of refractive index considered.

DE has managed to provide a solution to the problem which is slightly better than Needle for a number of layers which can be considered as quite high (29 layers). We do not claim here that DE is better than Needle, which makes no sense, but at least that it may produce solutions which can compete with Needle on the problem of designing optical filters - which is already a feat.

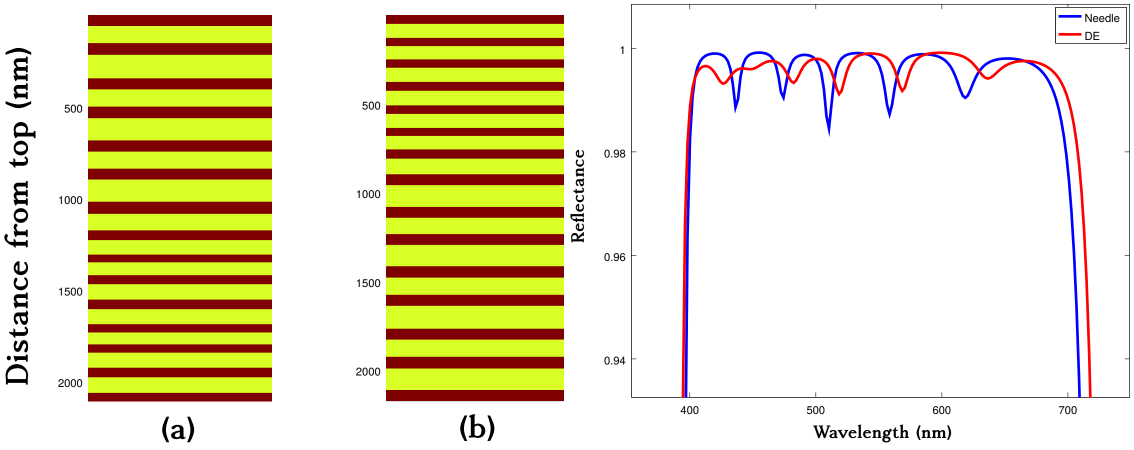

Figure 15. Left: (a) Structure generated using Needle[45], comporting 29 layers (b) Best structure found using DE for 29 layers. Right : Comparison of the two reflectance spectra showing that the dielectric mirror proposed by DE is actually slightly better than the structure proposed by Needle.

VIII. ANTIREFLECTIVE COATING FOR SILICON PHOTOVOLTAICS

For the design of the anti-reflective (AR) coating, we relied on our scattering matrix method for multilayers, Moosh[13], for computing the conversion efficiency. We used the standard material parameters that come along with the program. The objective function is simply taken as one minus the conversion efficiency, computed by assuming that each photon (in the 375-750 nm range) that is absorbed in the active (amorphous silicon) layer in converted into an electron-hole pair and then collected. We then compare the short-circuit current this procedure yields with the theoretical maximum short-circuit current that can be reached when all the photons produce a pair and are then collected (always in the 375-750 range for which amorphous silicon may produce electron-holes pairs). With this procedure, we assure that the objective function is comprised between 0 and 1.

We ran optimizations on two cases, and two cases only so far (i) a case where the amorphous silicon layer is very thin (89 nm), that we studied before[35], and for which we known that for 89 nm we have a marked resonance in the absorption spectrum that makes the layer more absorbent and (ii) a case where the layer is, on the contrary, quite thick (10 μm) in order to be in a more conventional framework for photovoltaics. Having seen that many results, for multilayers, present a maximum index contrast, and that the optimization was usually much more successful when the contrast was imposed (as seen above), we have done so right away. We thus excluded all the solutions that could have looked like index gradients, solutions some of us have been studying though in another context[7]. We were at the time simply curious to see what the algorithms could yield. Again, the results we have presented are our very first runs, and this is enough to demonstrate that elegant and surprising solutions can emerge through optimization. The

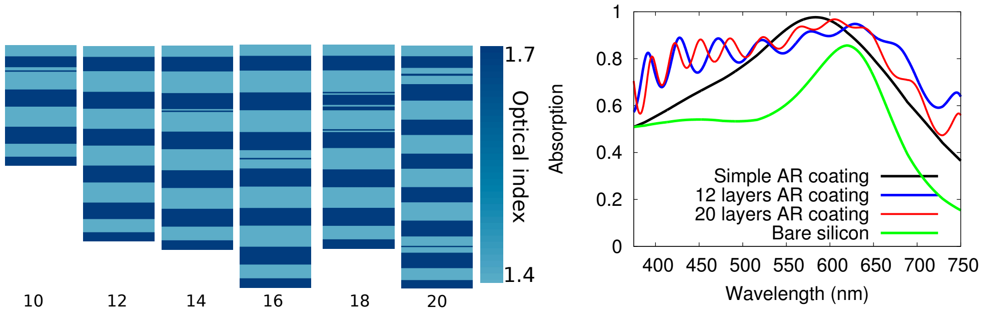

Figure 16. Optimal structures produced by the algorithms for a 89 nm thick amorphous silicon layer(left) and absorption spectra for a few chosen designs (right)

rest is left for future works.

For the thin amorphous silicon layer, the resulting structures and the corresponding absorption spectrum are shown on Fig. 16. In order to be fair, we compared the performances of the structured AR coating with 12 and 20 layers with a one-layer AR coating. For the latter, the optimal characteristics are well known. The thickness of the layer has to be chosen so that it is equal to a fourth of the central operation wavelength. We have taken here a layer with an optical index of 1.7 (more efficient than a 1.4 index coating) and a thickness of 95.6 nm, corresponding to a central operation wavelength of 650 nm.

We underline that, because the layer itself is resonant, the optimum number of layers seems to be 12. We have computed the conversion efficiency of the structure for different numbers of layers and came to the conclusion that although the change in the conversion efficiency are small, it is at its highest for 4 or 5 periods and thus 10 or 12 layers. That is why the results of the optimization for 14 and 16 layers looks exactly like the 12 layer AR coating (the thicknesses of some layers are clearly extremely small so that they don't contribute to the optical response).

For a thicker silicon layer, the results of the optimization are much more clear because the layer is not resonant any more. The advantage brought by the structured AR coating is however smaller compared to the simple AR coating (the same 95.6 nm thick layer as above). The results are clearly Bragg mirrors with modified edges. It is striking to see that for 12 layers, the optimum structure is the same for the thick or the thin silicon layer.

Of course, the case we have treated here is simple. Again, our point was not to optimize a real structure with perfectly accurate material parameters (in general the simple AR coatings are less efficient for crystalline silicon than what is shown here and more complex AR coatings are thus even more relevant[14]). Here, obviously, the algorithms consistently point towards an unexpected class of AR coatings rather than very different solutions for different thicknesses of the active silicon layer or different numbers of layers

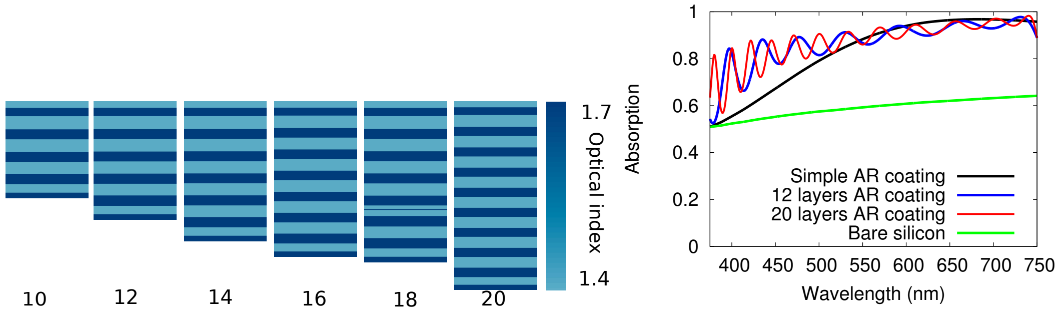

Figure 17. Optimal structures produced by the algorithms for a 10 μm thick amorphous silicon layer(left) and absorption spectra for a few chosen designs (right)

Structure type	Short-circuit current Jsc	**Conversion efficiency CE**
89 nm layer	12.859	0.55778
89 nm layer & AR coating	17.075	0.74067
10 μm layer	13.759	0.59632
10 μm layer & AR coating	19.991	0.86639

Table VII. Performances for the photovoltaic structures we use as a reference: a 89 nm thick and a 10 μm thick amorphous silicon layer, bare and covered with a $\lambda/4$ anti-reflective coating (the central wavelength λ being chosen to be 650 nm).

in the coating. This is what makes the results very promising – we can be sure that this method, applied to other problems in photonics, will be able to produce efficient, regular and intelligible structures.

[1] E. Balsa-Canto, J. R. Banga, J. A. Egea, A. Fernandez-Villaverde, and G. M. de Hijas-Liste. *Global Optimization in Systems Biology: Stochastic Methods and Their Applications*, pages 409–424. Springer New York, New York, NY,

**Number of layers**	**89 thick silicon layer**		**10 μm silicon layer**	
	Short-circuit current Jsc	Conversion efficiency CE	Short-circuit current Jsc	Conversion efficiency CE
10	19.011	0.79615	21.001	0.87949
12	19.062	0.79827	21.032	0.88080
14	19.042	0.79746	21.056	0.88181
16	19.047	0.79768	21.075	0.88261
18	19.021	0.79655	21.072	0.88245
20	19.057	0.79808	21.100	0.88364

Table VIII. Performances of the different multilayered anti-reflective coatings.

2012.

[2] J. R. Banga. Optimization in computational systems biology. *BMC systems biology*, 2(1):1, 2008.

[3] J. Beaulieu, C. Gagneé, and M. Parizeau. Lens system design and re-engineering with evolutionary algorithms. In *Proceedings of the 4th Annual Conference on Genetic and Evolutionary Computation*, pages 155–162. Morgan Kaufmann Publishers Inc., 2002.

[4] V. Berthier. Progressive differential evolution on clustering real world problems. In *International Conference on Artificial Evolution (Evolution Artificielle)*, pages 71–82. Springer, 2015.

[5] J. Bibai, P. Savéant, M. Schoenauer, and V. Vincent. An Evolutionary Metaheuristic for Domain-Independent Satisficing Planning. In R. Brafman, H. Geffner, J. Hoffmann, and H. Kautz, editors, *20th International Conference on Automated Planning and Scheduling-ICAPS2010*, pages 15–25, Toronto, Canada, May 2010. AAAI Press.

[6] D. Bratton and J. Kennedy. Defining a standard for particle swarm optimization. In *IEEE Swarm Intelligence Symposium*, pages 120–127, 2007.

[7] E. Centeno, A. Farahoui, R. Smaali, A. Bousquet, F. Réveret, O. Teytaud, A. Moreau, et al. Ultra thin anti-reflective coatings designed using differential evolution. *arXiv preprint arXiv:1904.02907*, 2019.

[8] S. Chatterjee, M. Laudato, and L. A. Lynch. Genetic algorithms and their statistical applications: an introduction. *Computational Statistics & Data Analysis*, 22(6):633–651, 1996.

[9] M. Clerc. Beyond standard particle swarm optimisation. *IJSIR*, 1(4):46–61, 2010.

[10] M. Clerc and J. Kennedy. The particle swarm - explosion, stability, and convergence in a multidimensional complex space. *Evolutionary Computation, IEEE Transactions on*, 6(1):58–73, 2002.

[11] A. R. Conn, K. Scheinberg, and L. N. Vicente. *Introduction to Derivative-Free Optimization*. Society for Industrial and Applied Mathematics, Philadelphia, PA, USA, 2009.

[12] S. Das and P. N. Suganthan. Differential evolution: A survey of the state-of-the-art. *IEEE Transactions on Evolutionary Computation*, 15(1):4–31, Feb 2011.

[13] J. Defrance, C. Lemaître, R. Ajib, J. Benedicto, E. Mallet, R. Pollès, J.-P. Plumey, M. Mihailovic, E. Centeno, C. Ciracì, D. Smith, and A. Moreau. Moosh: A numerical swiss army knife for the optics of multilayers in octave/matlab. *Journal of Open Research Software*, 4(1), 2016.

[14] A. Farhaoui, A. Bousquet, R. Smaali, A. Moreau, E. Centeno, J. Cellier, C. Bernard, R. Rapegno, F. Réveret, and E. Tomasella. Reactive gas pulsing sputtering process, a promising technique to elaborate silicon oxynitride multilayer nanometric antireflective coatings. *Journal of Physics D: Applied Physics*, 50(1):015306, 2016.

[15] M. Gallagher. Clustering problems for more useful benchmarking of optimization algorithms. In *Proceedings of the 10th International Conference on Simulated Evolution and Learning - Volume 8886*, SEAL 2014, pages 131–142, New York, NY, USA, 2014. Springer-Verlag New York, Inc.

[16] A. Gondarenko, S. Preble, J. Robinson, L. Chen, H. Lipson, and M. Lipson. Spontaneous emergence of periodic patterns in a biologically inspired simulation of photonic structures. *Physical review letters*, 96(14):143904, 2006.

[17] N. I. M. Gould, D. Orban, and P. L. Toint. Cuter and sifdec: A constrained and unconstrained testing environment, revisited. *ACM Trans. Math. Softw.*, 29(4):373–394, 2003.

[18] G. Granet and B. Guizal. Efficient implementation of the coupled-wave method for metallic lamellar gratings in

tm polarization. *JOSA A*, 13(5):1019–1023, 1996.

[19] C. H. Granier, F. O. Afzal, S. G. Lorenzo, M. Reyes Jr, J. P. Dowling, and G. Veronis. Optimized aperiodic multilayer structures for use as narrow-angular absorbers. *Journal of Applied Physics*, 116(24):243101, 2014.

[20] N. Hansen. *The CMA Evolution Strategy: A Comparing Review*, pages 75–102. Springer Berlin Heidelberg, Berlin, Heidelberg, 2006.

[21] N. Hansen, A. Auger, R. Ros, S. Finck, and P. Posik. Comparing Results of 31 Algorithms from the Black-Box Optimization Benchmarking BBOB-2009. In *ACM-GECCO Genetic and Evolutionary Computation Conference*, Portland, United States, July 2010. pp. 1689-1696.

[22] N. Hansen, A. Auger, R. Ros, S. Finck, and P. Pošík. Comparing results of 31 algorithms from the black-box optimization benchmarking bbob-2009. In *Proceedings of the 12th Annual Conference Companion on Genetic and Evolutionary Computation*, GECCO '10, pages 1689–1696, New York, NY, USA, 2010. ACM.

[23] N. Hansen, A. Auger, R. Ros, S. Finck, and P. Pošík. Comparing results of 31 algorithms from the black-box optimization benchmarking bbob-2009. In *Proceedings of the 12th Annual Conference Companion on Genetic and Evolutionary Computation*, GECCO '10, pages 1689–1696, New York, NY, USA, 2010. ACM.

[24] N. Hansen and A. Ostermeier. Completely derandomized self-adaptation in evolution strategies. *Evolutionary Computation*, 11(1), 2003.

[25] C. Z. Janikow and Z. Michalewicz. An experimental comparison of binary and floating point representations in genetic algorithms. In *ICGA*, pages 31–36, 1991.

[26] C. Kane and M. Schoenauer. Topological optimum design using genetic algorithms. *Control and Cybernetics*, 25:1059–1088, 1996.

[27] C. Kavka, P. Roggero, and M. Schoenauer. Evolution of voronoi based fuzzy recurrent controllers. In *Proceedings of the 7th Annual Conference on Genetic and Evolutionary Computation*, GECCO '05, pages 1385–1392, New York, NY, USA, 2005. ACM.

[28] J. Kennedy and R. C. Eberhart. Particle swarm optimization. In *Proceedings of the IEEE International Conference on Neural Networks*, pages 1942–1948, 1995.

[29] P. Lalanne and G. M. Morris. Highly improved convergence of the coupled-wave method for tm polarization. *JOSA A*, 13(4):779–784, 1996.

[30] A. Macleod. The quarterwave stack: 1. early history. *Society of Vaccum Coaters Bulletin*, pages 22–27, 2012.

[31] A. Macleod. The quarterwave stack: 2. properties. *Society of Vaccum Coaters Bulletin*, pages 16–21, 2012.

[32] A. Macleod. The quarterwave stack: 3. a building block. *Society of Vaccum Coaters Bulletin*, pages 16–21, 2012.

[33] H. A. Macleod. *Thin-film optical filters.* CRC press, 2001.

[34] S. Martin, J. Rivory, and M. Schoenauer. Synthesis of optical multilayer systems using genetic algorithms. *Applied Optics*, 34(13):2247–2254, 1995.

[35] A. Moreau, R. Smaali, E. Centeno, and C. Seassal. Optically optimal wavelength-scale patterned ito/zno composite coatings for thin film solar cells. *Journal of Applied Physics*, 111(8):083102, 2012.

[36] J. A. Nelder and R. Mead. A Simplex Method for Function Minimization. *The Computer Journal*, 7(4):308–313, Jan. 1965.

[37] K. E. Parsopoulos and M. N. Vrahatis. Parameter selection and adaptation in unified particle swarm optimization. *Mathematical and Computer Modelling*, 46(1-2):198–213, 2007.

[38] I. Rechenberg. *Evolutionsstrategie Optimierung technischer Systeme nach Prinzipien der biologischen Evolution*. Friedrich Frommann Verlag, Stuttgart-Bad Cannstatt, 1973.

[39] M. F. Schubert, F. W. Mont, S. Chhajed, D. J. Poxson, J. K. Kim, and E. F. Schubert. Design of multilayer antireflection coatings made from co-sputtered and low-refractive-index materials by genetic algorithm. *Optics express*, 16(8):5290–5298, 2008.

[40] Y. Shi and R. C. Eberhart. A Modified Particle Swarm Optimizer. In *Proceedings of IEEE International Conference on Evolutionary Computation*, pages 69–73, Washington, DC, USA, May 1998. IEEE Computer Society.

[41] R. Storn and K. Price. Differential evolution – a simple and efficient heuristic for global optimization over continuous spaces. *J. of Global Optimization*, 11(4):341–359, Dec. 1997.

[42] P. N. Suganthan, N. Hansen, J. J. Liang, K. Deb, Y.-P. Chen, A. Auger, and S. Tiwari. Problem definitions and evaluation criteria for the cec 2005 special session on real-parameter optimization. Technical Report AND KanGAL Report #2005005, IIT Kanpur, India, 2005.

[43] O. Teytaud and S. Gelly. DCMA: yet another derandomization in covariance-matrix-adaptation. In H. Lipson, editor, *Genetic and Evolutionary Computation Conference, GECCO 2007, Proceedings, London, England, UK, July 7-11, 2007*, pages 955–963. ACM, 2007.

[44] S. Thibault, C. Gagné, J. Beaulieu, and M. Parizeau. Evolutionary algorithms applied to lens design: Case study and analysis. In *Proc. SPIE*, volume 5962, pages 66–76, 2005.

[45] A. V. Tikhonravov, M. K. Trubetskov, and G. W. DeBell. Application of the needle optimization technique to the design of optical coatings. *Applied optics*, 35(28):5493–5508, 1996.

[46] I. C. Trelea. The particle swarm optimization algorithm: convergence analysis and parameter selection. *Information Processing Letters*, 85(6):317 – 325, 2003.

[47] P. Vukusic, J. Sambles, C. Lawrence, and R. Wootton. Quantified interference and diffraction in single morpho butterfly scales. *Proceedings of the Royal Society of London B: Biological Sciences*, 266(1427):1403–1411, 1999.

[48] J.-M. Yang, J.-T. Horng, C.-J. Lin, and C.-Y. Kao. Optical coating designs using the family competition evolutionary algorithm. *Evolutionary Computation*, 9(4):421–443, 2001.

[49] J.-M. Yang and C.-Y. Kao. An evolutionary algorithm for the synthesis of multilayer coatings at oblique light incidence. *Journal of lightwave technology*, 19(4):559, 2001.

[50] M. Zambrano-Bigiarini, M. Clerc, and R. Rojas. Standard particle swarm optimisation 2011 at cec-2013: A baseline for future pso improvements. In *IEEE Congress on Evolutionary Computation*, pages 2337–2344. IEEE, 2013.
